# Effects of enriched-potassium diet on cardiorespiratory outcomes in experimental non-ischemic chronic heart failure

**DOI:** 10.1186/s40659-021-00365-z

**Published:** 2021-12-24

**Authors:** Karla G. Schwarz, Katherin V. Pereyra, Camilo Toledo, David C. Andrade, Hugo S. Díaz, Esteban Díaz-Jara, Domiziana Ortolani, Angélica Rios-Gallardo, Paulina Arias, Alexandra Las Heras, Ignacio Vera, Fernando C. Ortiz, Nibaldo C. Inestrosa, Carlos P. Vio, Rodrigo Del Rio

**Affiliations:** 1 Laboratory of Cardiorespiratory Control, Department of Physiology, Pontificia Universidad Católica de Chile, Santiago, Chile; 2 Centro de Excelencia en Biomedicina de Magallanes (CEBIMA), Universidad de Magallanes, Punta Arenas, Chile; 3 Centro de Fisiología y Medicina de Altura, Departamento Biomédico, Facultad de Ciencias de la Salud, Universidad de Antofagasta, Antofagasta, Chile; 4 Mechanisms of Myelin Formation and Repair Laboratory, Instituto de Ciencias Biomédicas, Facultad de Ciencias de Salud, Universidad Autónoma de Chile, Santiago, Chile; 5 Facultad de Medicina y Ciencia, Universidad San Sebastián, Santiago, Chile; 6 Centro de Envejecimiento y Regeneración (CARE), Pontificia Universidad Católica de Chile, Santiago, Chile

**Keywords:** Heart failure, Potassium supplemented diet, Autonomic imbalance, Breathing disorders, Chemoreflex function

## Abstract

**Background:**

Chronic heart failure (CHF) is a global health problem. Increased sympathetic outflow, cardiac arrhythmogenesis and irregular breathing patterns have all been associated with poor outcomes in CHF. Several studies showed that activation of the renin-angiotensin system (RAS) play a key role in CHF pathophysiology. Interestingly, potassium (K^+^) supplemented diets showed promising results in normalizing RAS axis and autonomic dysfunction in vascular diseases, lowering cardiovascular risk. Whether subtle increases in dietary K^+^ consumption may exert similar effects in CHF has not been previously tested. Accordingly, we aimed to evaluate the effects of dietary K^+^ supplementation on cardiorespiratory alterations in rats with CHF.

**Methods:**

Adult male Sprague–Dawley rats underwent volume overload to induce non-ischemic CHF. Animals were randomly allocated to normal chow diet (CHF group) or supplemented K^+^ diet (CHF+K^+^ group) for 6 weeks. Cardiac arrhythmogenesis, sympathetic outflow, baroreflex sensitivity, breathing disorders, chemoreflex function, respiratory–cardiovascular coupling and cardiac function were evaluated.

**Results:**

Compared to normal chow diet, K^+^ supplemented diet in CHF significantly reduced arrhythmia incidence (67.8 ± 15.1 vs. 31.0 ± 3.7 events/hour, CHF vs. CHF+K^+^), decreased cardiac sympathetic tone (ΔHR to propranolol: − 97.4 ± 9.4 vs. − 60.8 ± 8.3 bpm, CHF vs. CHF+K^+^), restored baroreflex function and attenuated irregular breathing patterns. Additionally, supplementation of the diet with K^+^ restores normal central respiratory chemoreflex drive and abrogates pathological cardio-respiratory coupling in CHF rats being the outcome an improved cardiac function.

**Conclusion:**

Our findings support that dietary K^+^ supplementation in non-ischemic CHF alleviate cardiorespiratory dysfunction.

**Supplementary Information:**

The online version contains supplementary material available at 10.1186/s40659-021-00365-z.

## Background

Chronic heart failure (CHF) affecting ~ 26 million people worldwide [1], represent the leading cause of hospital admissions in people over 65 years old [2]. One form of CHF is due to the loss of ventricular mass following ischemic events which trigger cardiac systolic dysfunction (< 40 ejection fraction, EF). Contrarily, non-ischemic CHF is characterized by normal EFs and marked impairments in cardiac diastolic function [3]. Notably, the prevalence of non-ischemic CHF is increasing in the worldwide population and mortality remains high. The later evidence the lack of effective means to help in the management of non-ischemic heart disease [3]. Despite the progress in therapeutic strategies, non-ischemic CHF prevalence is increasing over time and mortality remains high due to the greater number of both elderly people and comorbidities associated with CHF (i.e. hypertension, obesity, and coronary artery disease) [3, 4]. Therefore, novel treatments for non-ischemic CHF have become a global health priority [1]. Pathophysiological hallmarks of CHF patients include autonomic dysfunction characterized by cardiac sympathetic overactivity and parasympathetic withdrawal [5, 6], alterations in heart rate variability, and reduction of cardiac baroreflex sensitivity [7]. Importantly, enhanced cardiac sympathetic drive is a potent trigger for cardiac arrhythmogenesis which may lead to increase the risk of decompensation and mortality in CHF [8]. Furthermore, almost ~ 50% of CHF patients display alterations in resting breathing patterns (i.e. apneas, hypopneas, periodic breathing) [9] that add more stress to the heart through chemoreflex activation of the sympathetic nervous system. Conversely, increased chemoreflex activation also promotes the development of altered breathing patterns then creating a vicious cycle that compromises further deterioration in cardiac function [10].

Several molecular mechanisms have been pointed out to contribute to CHF progression. However, a growing body of evidence suggests that chronic activation of the renin-angiotensin-system (RAS) is closely related with CHF progression [11]. Indeed, Angiotensin II (AngII)-induced reactive oxygen species (ROS) production in the central nervous systems enhances sympathetic outflow in CHF [11, 12]. Furthermore, increases in systemic and brain AngII levels in CHF has also been reported. AngII-derived ROS are mainly originated through activation of nicotinamide adenine dinucleotide phosphate (NADPH) oxidase via AngII receptor type I (AT_1_R) [13, 14]. Importantly, previous studies shown that non-ischemic CHF rats display enhanced NADPH oxidase activation and superoxide production in the rostral ventrolateral medulla (RVLM), a major site for sympathetic regulation [15–17]. Therefore, strategies intended to reduce AngII-derived ROS and their well-known pathophysiological consequences in CHF may contribute to the control of cardiovascular and respiratory alterations.

Potassium (K^+^) supplemented or enriched diets have been proposed as a feasible complementary clinical indication to prevent major detrimental cardiovascular events, particularly for the management of arterial blood pressure in human and experimental hypertension [18–21]. Indeed, K^+^ intake prevents the induction of RAS, improves sodium excretion and blood pressure regulation by modulating renal sympathetic activity [22–24]. Furthermore, a cardioprotective effect of dietary K^+^ has also been described since subtle increases in K^+^ inhibits NADPH oxidase activity and reduced ROS formation [25, 26]. This evidence supports that dietary K^+^ supplementation may have beneficial physiological effects in disease states characterized by RAS activation and sympathoexcitation [27]. Importantly, western diets are characterized by K^+^ deficiency and excessive sodium (Na^+^) intake and have been largely linked to the development/aggravation of cardiovascular diseases [28–30].

Together, evidence support the notion that dietary K^+^ supplementation may offer benefit in the setting of CHF; however, to our knowledge there are no comprehensive studies showing if increases in daily K^+^ intake may help improving cardiovascular and respiratory function in CHF. Accordingly, we aimed to determine the effect of dietary K^+^ supplementation on sympathetic and parasympathetic outflow, baroreflex sensitivity, cardiac arrhythmogenesis, breathing disorders, chemoreflex function, respiratory–cardiovascular coupling and cardiac function in experimental non-ischemic CHF.

## Methods

### Ethical considerations and animals

Experiments were performed on 15 male Sprague–Dawley rats housed in a controlled temperature environment with light/dark cycle of 12 h and water-food ad libitum (see Additional file 1: Fig. S1 for experimental timeline). The protocol was approved by the Ethical-Scientific Committee of the Facultad de Ciencias Biológicas, Pontificia Universidad Católica de Chile (Protocol ID 170710022), and was conducted in accordance with the National Institutes of Health (NIH) Guide for the Care and Use of Laboratory Animals. All animals were humanely euthanized with an overdose of sodium pentobarbital (100 mg kg^−1^ ip).

### Experimental non-ischemic CHF

Non-ischemic chronic heart failure (CHF) was produced by the surgical creation of an arteriovenous shunt between the abdominal aorta and the cava vein to induced chronic volume overload [16, 17, 31–34]. Under anesthesia (induction with 5% of isoflurane and 2% for maintenance, balance with O_2_) laparotomy was performed. Under a dissection microscope, abdominal aorta and inferior cava vein were carefully exposed and the abdominal aorta was punctured with an 18-gauge needle until the adjacent cava vein was reached. A drop of Hystoacril (Braun) tissue glue was used to seal the aortic puncture. Fistula patency was visually confirmed by the presence of arterial pulsatile blood flow towards the venous circulation. Finally, abdominal cavity was closed in layers with absorbable 4–0 Vycril suture (Braun). Antibiotic (enrofloxacin 10 mg Kg^−1^ s.c.), analgesic (ketoprofen 5 mg Kg^−1^ s.c.) and saline (3 ml of 0.9% NaCl solution i.p.) were administrated post-surgery. Sham-operated rats underwent the exact same procedure with the only exception that no puncture was done in the aorta.

### Echocardiography

Under anesthesia (1.5–2% isoflurane balance with O_2_), transthoracic M-mode echocardiography (Mindray Z6 Vet) at week 2 and 8 post CHF surgery were performed. Recordings were made from the left parasternal short-axis view. An increase of 1.5-fold in the end diastolic volume (EDV) and stroke volume (SV) relative to sham condition were the criteria for CHF [16, 17, 31, 33, 34]. Left ventricle (LV) end diastolic and systolic diameter (LVEDD and LVESD, respectively) were measured from 3 consecutive cycles. LV end diastolic and systolic volume (LVEDV and LVESV, respectively) where derived from Teicholz method ([LVESV = 7*ESD^3^/(2.4 + ESD)] and [LVEDV = 7*EDD^3^/(2.4 + EDD)]. The ejection fraction (EF) and fractional shortening (FS) were calculated from left ventricle volumes and diameters, respectively. The cardiac output (CO) is the product from SV and heart rate (HR).

### Potassium diet supplementation

Rats were randomly assigned to Sham, CHF and CHF+K^+^ groups. Sham and CHF received standard chow diet (Prolab® RMH3000 5P00/0.9% K^+^) while CHF+K^+^ received the same formula but supplemented with 3% K^+^ (Prolab® RMH3000 5P00/2% K^+^ and 1% KCl in the drinking water) for 6 weeks as previously described [22, 23]. Combination of K^+^ supplementation in both chow and tap water has been shown to increase tolerability to high-salt diets [35]. Body weight gain, daily food and water intake were registered in all groups and averaged. See Additional file 1: Table S1 for complete diets composition.

### Telemetry implant for blood pressure and heart rate measurements

Radio-telemetry pressure transducer (HD-S10, ADInstruments) were implanted 7 weeks after CHF or sham surgery. Rats under isoflurane anesthesia underwent a skin incision to expose and isolate femoral artery. The tip of a pressure transducer was guided into the femoral artery and the HD-S10 digital transmitter was placed subcutaneously. Then, rats received antibiotic (enrofloxacin 10 mg Kg^−1^ s.c.) and analgesic (ketoprofen 5 mg Kg^−1^ s.c.). After 1 week of recovery, arterial blood pressure (BP) was measured in conscious rats [31, 34]. Mean arterial blood pressure (MABP), systolic blood pressure (SBP), diastolic blood pressure (DBP) and pressure pulse (PP) were derived from BP signal. Heart rate (HR) was derived from dP/dt signal of BP.

### Spontaneous baroreflex

Telemetry recording was used to analyze change in spontaneous baroreflex 8 weeks after CHF induction. MABP and HR were derived from BP signal. Baroreflex sequences where changes in HR (ΔHR) where associated with changes in MABP (ΔMABP) were used to estimate spontaneous baroreflex function. The up sequences were analyzed apart from down sequences. Then values were plotted, and linear regression was calculated for each animal [36].

### Sympatho-vagal balance

Cardiac sympathetic-vagal balance was evaluated by effects of propranolol (1 mg Kg^−1^ i.p.), a non-selective β-adrenergic receptor antagonist, and atropine injection (1 mg Kg^−1^ i.p.), a muscarinic receptor antagonist on HR in conscious rats. Changes in HR in response to propranolol were used as an indicator of sympathetic tone and HR responses to atropine were used as an indicator of parasympathetic tone. ∆HR represent the change in HR respect from baseline HR [16, 33].

### Arrhythmia incidence

Irregular heartbeats were visually inspected and counted as previously described [33]. Arrhythmias were defined as premature or delayed beats with changes greater than 3 standard deviations (SD) from the mean beat-to-beat interval duration. The arrhythmia index was expressed as events/hour.

### Resting breathing patterns and chemoreflex function

Unrestrained whole-body plethysmography (Emka Technologies) was used to record resting breathing (RB) and chemoreflex function. RB was recorded during 2 h at room air. Central and peripheral chemoreflex function was evaluated during 10 min of hypercapnia (7% F_i_CO_2_/balance with N_2_) and hypoxia (10% F_i_O_2_/balance with N_2_) gas challenges, respectively. An interval of 20 min in normoxia separated the two stimuli. Tidal volume (V_T_ [ml 100 g^−1^]), respiratory frequency (R_f_ [breath min^−1^]) and minute volume (V_E_ [ml min^−1^ 100 g^−1^]) was obtained using ecgAUTO post-processing software (Emka Technologies). Regularity of breathing pattern was evaluated using Poincare plots of the breath-to-breath interval variability from 6 independent and random intervals of ~ 400 breaths. Coefficient of variation of V_T_ was also calculated to evaluate oscillations in ventilatory cycles. Irregularity score (IS) was evaluated using the follow equation: 100*(T_TOTn_−T_TOTn-1_)/T_TOTn-1_ for the nth respiratory cycle as described previously [34].

The hypercapnic ventilatory response (HCVR) was obtained by determining the slope on the V_E_ response between F_i_CO_2_ 0.03% and F_i_CO_2_ 7%. The hypoxic ventilatory response (HVR) was obtained by calculating the slope between V_E_ and F_i_O_2_ 21% and F_i_O_2_ 10%. All experiments were conducted at ambient temperature in daily time.

Breathing disorders including apneas (cessation in ventilation for at least 3 consecutive respiratory cycles) and hypopneas (reduction in V_T_ under 50% of normal breathing amplitude for at least 3 consecutive respiratory cycles) were scored from 1 h of RB recordings [34].

### Cardiorespiratory coupling

Several studies showed that coupling between respiratory and cardiovascular function promotes sympathoexcitation and perpetuates the generation of breathing disorders. Accordingly, we analyzed the effects of rising dietary K^+^ levels on cardiorespiratory coupling in the setting of non-ischemic CHF. Accordingly, coherence between V_T_ and SBP were evaluated through MATLAB routine (MathWorks) as described previously [31, 34, 37, 38]. Welch’s over-lapped segment averaging method were used to calculate auto- and cross-spectral estimates from 5 min recordings. Every variable underwent Fast Fourier transform (FFT). Respiratory signal oscillations were taken as the input and BP as the output for coherence analysis. Cut-off to evaluate the magnitude of mean square coherence was 0.015 Hz centered at the frequency of maximum spectral peak of V_T_ in the very low frequency domain (vLF: 0.01–0.25 Hz). Positive interaction between ventilation and BP were obtained from square coherence values over 0.5 (range of square coherence is 0–1).

### Cardiac function assessment

LV function was evaluated using a pressure–volume (PV) conductance catheter (SPR-869, Millar) [33, 39]. Under anesthesia (α-chloralose and urethane, 800 mg Kg^−1^ and 40 mg Kg^−1^, respectively) a conductance catheter was introduced into right carotid artery and advance toward LV chamber. A laparotomy was performed to visualized arteriovenous fistula and then perform a cava vein occlusion. PV-loops were recorded for 1 h and fifteen to twenty PV loops were used to calculate hemodynamic parameters: cardiac output (CO), ejection fraction (EF), end diastolic pressure (EDP), end systolic pressure (ESP), Parameters dependent of load were: dP/dt_max_, dP/dt_min_. Load-independent parameters: end-systolic pressure volume relationship (ESPVR) and end-diastolic pressure volume relationship (EDPVR) were also calculated. Data was processed using the PV-loop module of LabChart v7.3.8 software (ADInstruments).

### [Na^+^] and [K^+^] systemic concentrations

Under anesthesia α-chloralose/urethane anesthesia, arterial blood samples were obtained from the abdominal aorta using a 3 ml syringe. A drop of arterial blood was immediately analyzed (iSTAT1 CG8+ , Abbott) at the end of cardiac function assessment. Ionic concentration (K^+^ and Na^+^) of arterial blood was analyzed.

### Statistical analysis

Data is expressed as mean ± standard error mean (SEM). Data is shown as min to max box and whiskers plot in figures. One-way or two-way ANOVA, depending on data structure, was employed to evaluate differences between groups following by Holm-Sidak post hoc test. P < 0.05 was consider as statistically significant.

## Results

### K^+^ supplementation decreases arrhythmias and restores normal autonomic and baroreflex function in CHF

**Fig. 1:**
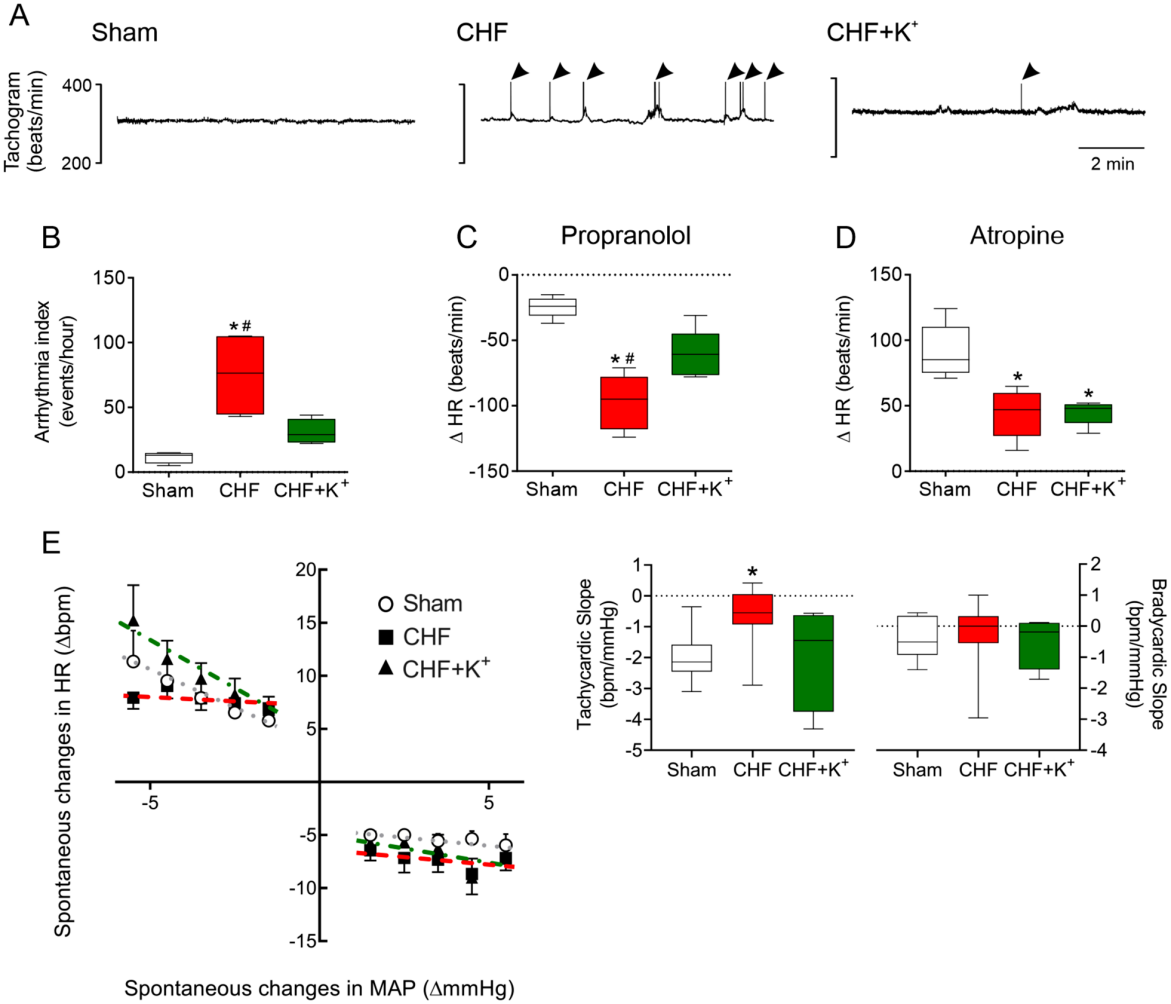
Dietary K^+^ supplementation decreases arrhythmia incidence, cardiac sympathetic tone and improves spontaneous baroreflex in CHF rats. **A** Representative recording of heart rate (HR) tachograms obtained from one Sham rat, one CHF rat and one CHF+K^+^ rat. Arrowheads indicate arrhythmic events. Note that K^+^ supplemented diet reduces arrhythmic events in CHF. **B** Summary data showing arrhythmia index (events/hour). **C** Heart rate responses (ΔHR) after sympathetic blockade with propranolol (1 mg/Kg). **D** ΔHR after parasympathetic blockade with atropine (1 mg/Kg). **E** Baroreflex sensitivity (BRS) during spontaneous changes in HR and mean arterial pressure (MAP). Each dashed line represents the tachycardic or bradycardic slope. *P < 0.05 vs Sham, ^#^P < 0.05 vs CHF+K^+^. Holm Sidak post hoc after One-Way ANOVA, n = 5 rats per group

CHF rats showed an increase in the number of cardiac arrhythmias compared to Sham rats (67.8 ± 15.1 vs. 10.4 ± 2.1 events/hour, CHF vs. Sham, respectively; p < 0.05). CHF rats that received diet supplemented with K^+^ showed a significant reduction in the number of arrhythmias compared to CHF rats (31.0 ± 3.7 vs. 67.8 ± 15.1 events/hour, CHF+K^+^ vs. CHF, respectively; p < 0.05) (Fig. 1A, B). In agreement with previous investigations, CHF rats displayed a heightened cardiac sympathetic drive compared to Sham healthy rats (ΔHR to propranolol: − 97.4 ± 9.4 vs. − 24.5 ± 3.6 bpm, CHF vs. Sham, respectively; p < 0.05) and this was normalized by the enriched-K^+^ diet in CHF. Indeed, CHF+K^+^ rats showed a significant reduction in cardiac sympathetic tone compared to CHF rats (ΔHR to propranolol: − 60.8 ± 8.3 bpm vs. − 97.4 ± 9.4 bpm, CHF+K^+^ vs. CHF, respectively; p < 0.05) (Fig. 1C). In addition, CHF group displayed a decreased parasympathetic drive compared to Sham (ΔHR to atropine: 44.1 ± 8.3 vs. 91.2 ± 9.2 bpm, CHF vs. Sham, respectively; p < 0.05) and this was not changed by K^+^ supplementation in CHF (44.1 ± 8.3 bpm and 44.8 ± 4.1 bpm, CHF+K^+^ vs. CHF, respectively) (Fig. 1D). In addition, tachycardic baroreflex gain was reduced in rats with CHF compared to Sham rats (slope: − 0.7 ± 0.3 vs − 2.0 ± 0.3 bpm/mmHg, CHF vs. Sham, respectively; p < 0.05) and dietary K^+^ supplementation in CHF significantly improved baroreflex gain (Fig. 1E). Bradycardic baroreflex responses were undistinguishable between groups (Fig. 1E).

### Effects of K^+^ supplementation on breathing patterns in CHF

**Fig. 2:**
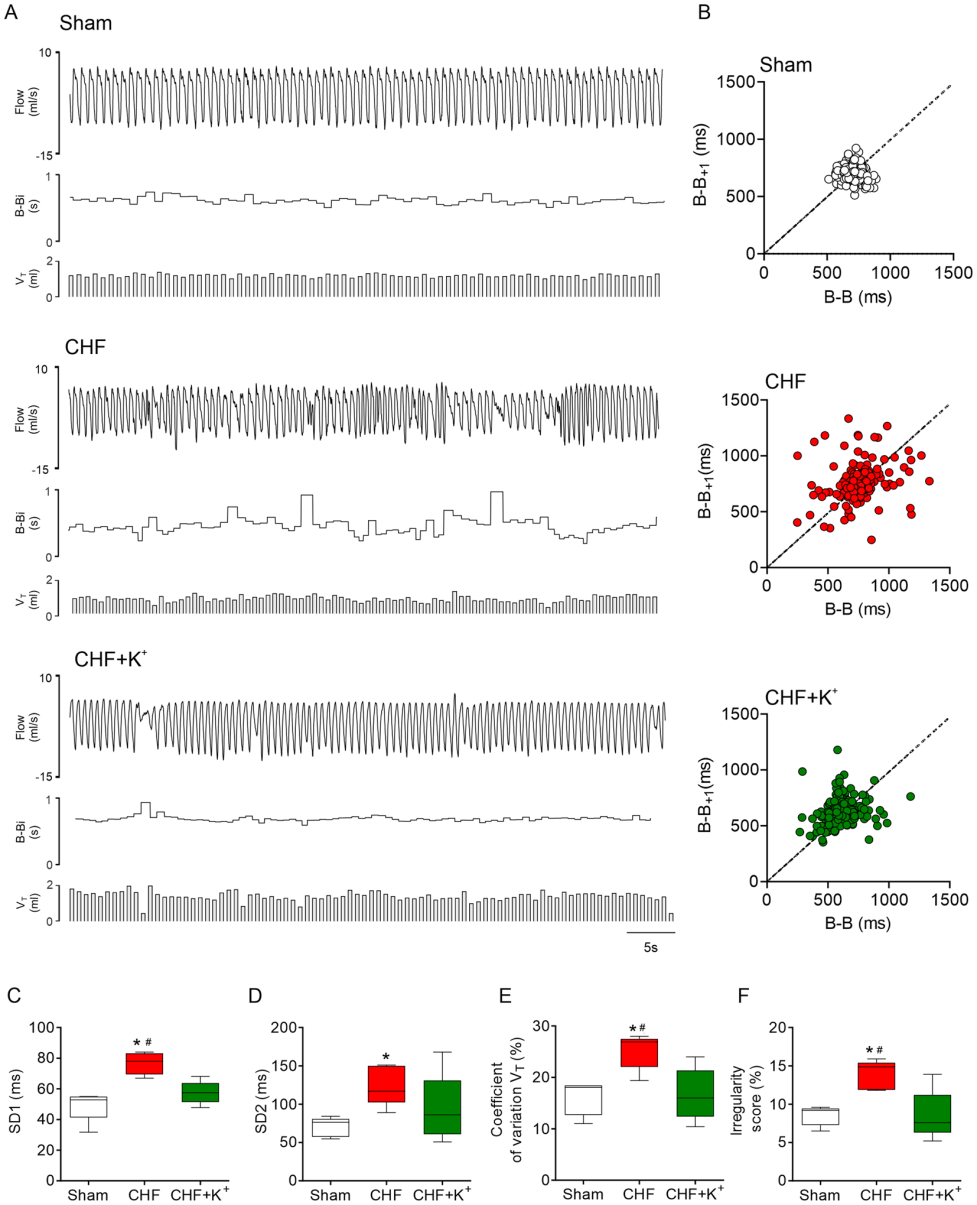
Daily dietary K^+^ supplementation improves breathing in CHF rats. **A** Representative ventilation recordings of ventilatory flow (ml/s), breath-to-breath interval (B-B_i_, s) and tidal volume (V_T_, ml) obtained from one Sham rat, one CHF rat and one CHF+K^+^ rat. **B** Representative Poincare plots showing B-B_i_ variability. **C**–**D** Summary data displaying SD1 and SD2 in all groups. Note that irregularity of B-B_i_ in CHF is markedly improve by dietary K^+^ supplementation. **E** Summary data showing changes in breathing irregularity score (%). **F** Coefficient of variation of V_T_ amplitudes (%). K^+^ supplemented diet significantly reduces V_T_ oscillations in CHF. *P < 0.05 vs Sham, ^#^P < 0.05 vs CHF+K^+^. Holm Sidak post hoc after One-Way ANOVA, n = 5 rats per group

Irregular resting breathing both in frequency and amplitude of respiration were observed in CHF rats compared to Sham (Fig. 2A). Indeed, interbreath interval variability was higher in CHF rats compared to Sham (SD1: 76.6 ± 3.3 vs. 49.1 ± 4.4 ms; SD2: 124.4 ± 11.6 vs. 70.6 ± 5.6 ms, CHF vs. Sham, respectively; p < 0.05) (Fig. 2B–D). Furthermore, the coefficient of variation (CV) of each tidal volume (V_T_) amplitude was significantly greater in CHF (25.2 ± 1.6 vs 16.4 ± 1.8%, CHF vs. Sham, respectively; p < 0.05) (Fig. 2E). Accordingly, CHF rats showed increased overall breathing irregularity score (IS) compared to Sham rats (13.9 ± 0.8 vs. 8.5 ± 0.6%, CHF vs. Sham, respectively; p < 0.05) (Fig. 2F). Daily dietary K^+^ supplementation in CHF rats improved breathing pattern regularity at rest compared to CHF untreated rats (Fig. 2A). Compared to CHF, rats that received K^+^ diet showed significant (CHF+K^+^ vs. CHF, p < 0.05) improvements in breath-to-breath interval variability (SD1: 57.5 ± 3.3 vs. 76.7 ± 3.2 ms) and V_T_ oscillations (CV of V_T_: 16.7 ± 2.3 vs. 25.5 ± 1.6%) and IS (8.5 ± 1.5 vs. 13.9 ± 0.8%) (Fig. 2B–F). Apneas and hypopneas were also increased in CHF compared to Sham (apneas: 4.8 ± 0.6 vs. 1.4 ± 0.4; hypopneas: 3.6 ± 0.5 vs. 2.4 ± 0.5 events/hour, respectively; p < 0.05). While K^+^ supplementation in CHF decreases the incidence of apneas this was not rise statistical significance (Additional file 1: Table S2). Frequency of sighs and post sigh apneas, as well as apnea duration, were not different between groups (Additional file 1: Table S2).

### ***K***^+^ supplemented diet decreases central chemoreflex drive in CHF

**Fig. 3:**
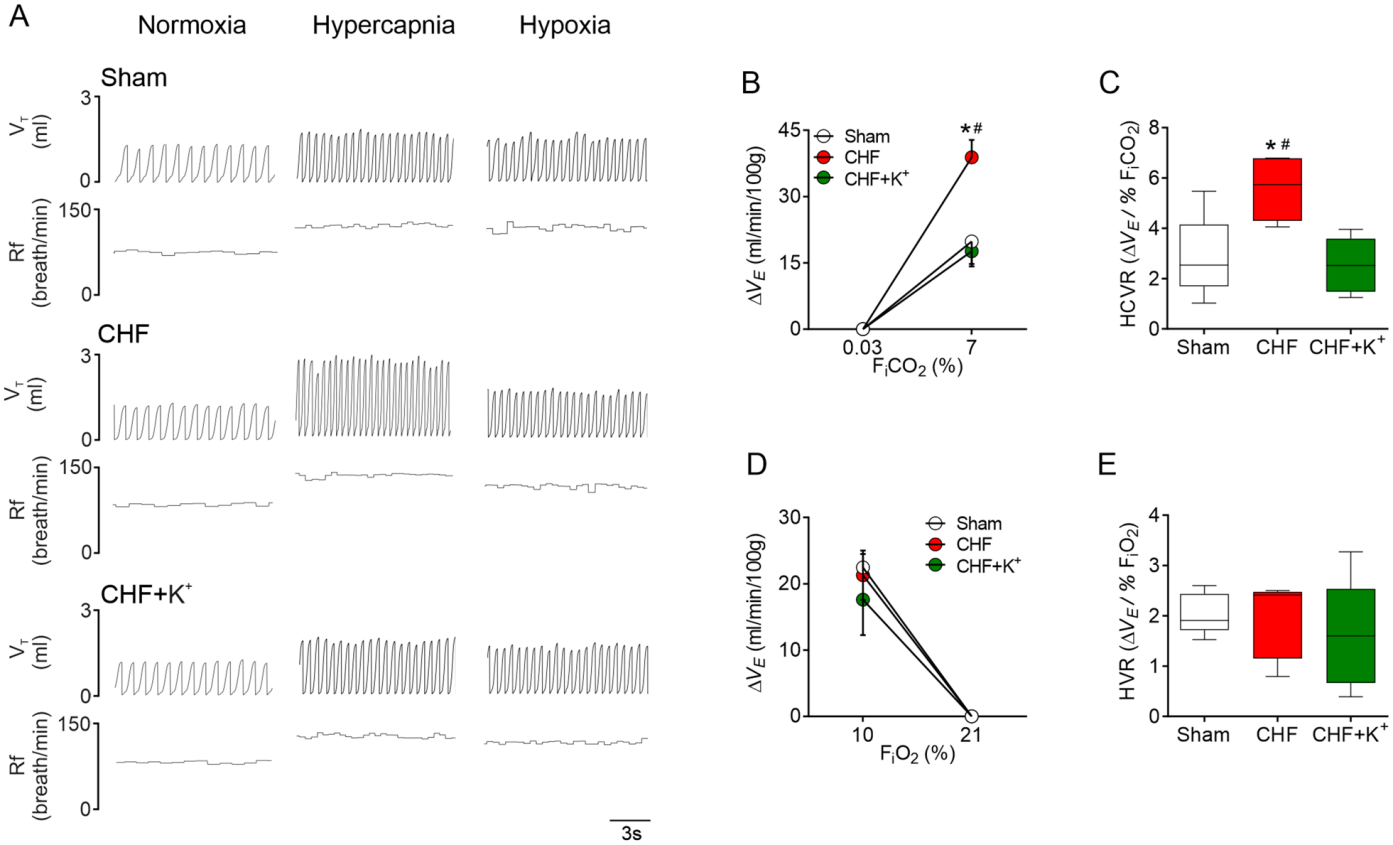
Central chemoreflex drive is normalized by K^+^ supplementation in CHF rats. **A** Representative recording of tidal volume (V_T_) and respiratory frequency (Rf) during normoxia (F_i_O_2_ 21%), hypercapnia (F_i_CO_2_ 7%) and hypoxia (F_i_O_2_ 10% in one Sham rat, one CHF rat and one CHF+K^+^ rat. **B**–**C** Summary data showing the magnitude (ΔV_E_, ml/min/100 g) and gain (ΔV_E_/%F_i_CO_2_) of the ventilatory response to hypercapnia (HCVR). Note that K^+^ supplementation totally restored normal HCVR in CHF rats. **D**–**E** Summary data showing the magnitude (ΔV_E_, ml/min/100 g) and gain (ΔV_E_/%F_i_O_2_) of the hypoxic ventilatory response (HVR). *P < 0.05 vs Sham, ^#^P < 0.05 vs CHF+K^+^. Holm Sidak post hoc after One-Way ANOVA, n = 5 rats per group

CHF rats showed an exaggerated central chemoreflex drive compared to Sham rats as evidenced by an enhanced ventilatory reflex response to hypercapnia (HCVR) (5.6 ± 0.6 vs. 2.8 ± 0.7 ΔV_E_/F_i_CO_2_ 7%, CHF vs. Sham, respectively; p < 0.05) (Fig. 3A–C). The observed potentiation in HCVR in CHF was abolished by dietary K^+^ supplementation (2.5 ± 0.5 vs. 5.6 ± 0.6 ΔV_E_/%F_i_CO_2_, CHF+K^+^ vs. CHF, respectively; p < 0.05) (Fig. 3C). No differences in the hypoxic ventilatory response (HVR) were found between groups (2.0 ± 0.2 vs. 1.9 ± 0.3 vs. 1.6 ± 0.5 ΔV_E_/%F_i_O_2_, Sham vs. CHF vs. CHF+K^+^, respectively) (Fig. 3D, E). Also, no significant changes in resting V_T_ nor in respiratory frequency in normoxia were found between experimental conditions (Additional file 1: Table S3).

### Effects of K^+^ supplementation in the diet on pathological cardiorespiratory coupling in CHF

**Fig. 4:**
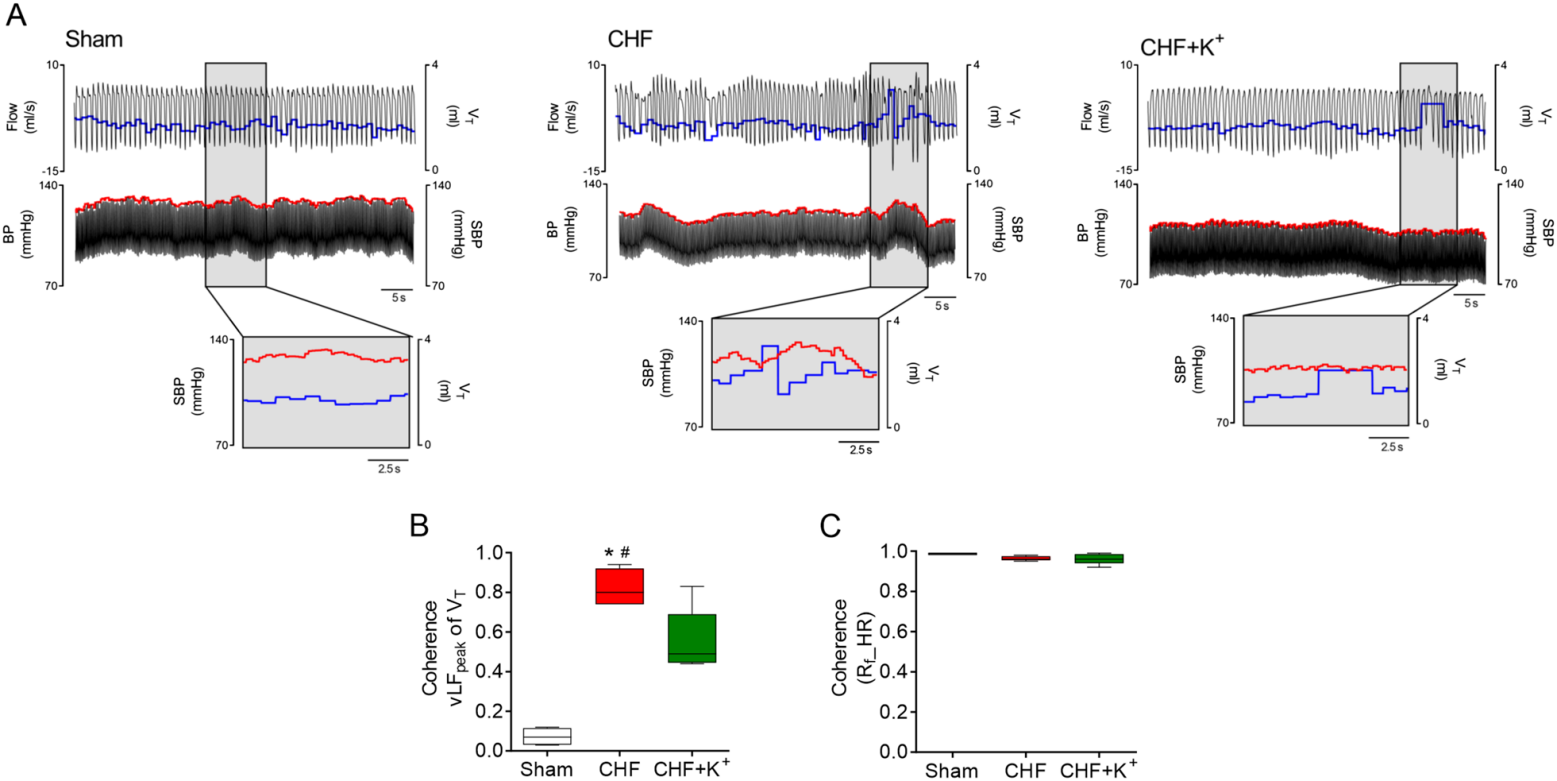
Dietary K^+^ supplementation attenuates cardiorespiratory coupling in CHF. **A** Representative traces of ventilatory flow (Flow, ml/s) and arterial blood pressure (BP, mmHg) in one Sham rat, one CHF rat and one CHF+K^+^ rat. Tidal volume (V_T_) is marked in blue while systolic blood pressure (SBP, mmHg) is shown in red. Note that in CHF rats, oscillations in ventilation are phase with increases in SBP, reflecting a positive interaction between signals. **B** Summary data showing the magnitude of coherence between V_T_ and SBP centered at the very low frequency (vLF) peak of V_T_. **C** Summary data showing coherence function between respiratory frequency (R_F_) and heart rate (HR). *P < 0.05 vs Sham, ^#^P < 0.05 vs CHF+K^+^. Holm Sidak post hoc after One-Way ANOVA, n = 5 rats per group

Rats with CHF exhibit cardiorespiratory coupling evidenced by the presence of significant coherence between V_T_ and SBP oscillations (Coherence: 0.8 ± 0.1 vs. 0.07 ± 0.1, CHF vs. Sham, respectively; p < 0.05). In addition, coherence in CHF was characterized by a positive phase angle (53.2 ± 18.6°) supporting that breathing oscillations and blood pressure were in phase with the cyclic changes in V_T_ amplitudes. CHF rats that received K^+^ supplemented diet displayed a decrease in the magnitude of coherence between V_T_ and SBP to levels comparable to the ones obtained in Sham rats (Coherence: 0.5 ± 0.1 vs*.* 0.8 ± 0.1, respectively; p < 0.05) (Fig. 4A, B). No differences in baseline SBP were found between groups (Additional file 1: Table S4). High coherence values between ventilation and heart rate were found in all groups (Fig. 4C). In summary, dietary K^+^ supplementation ameliorates the potentiated respiratory-sympathetic coupling in non-ischemic CHF rats, one pathophysiological hallmark of CHF disease.

### Effect of dietary K^+^ supplementation on echocardiographic parameters

**Fig. 5:**
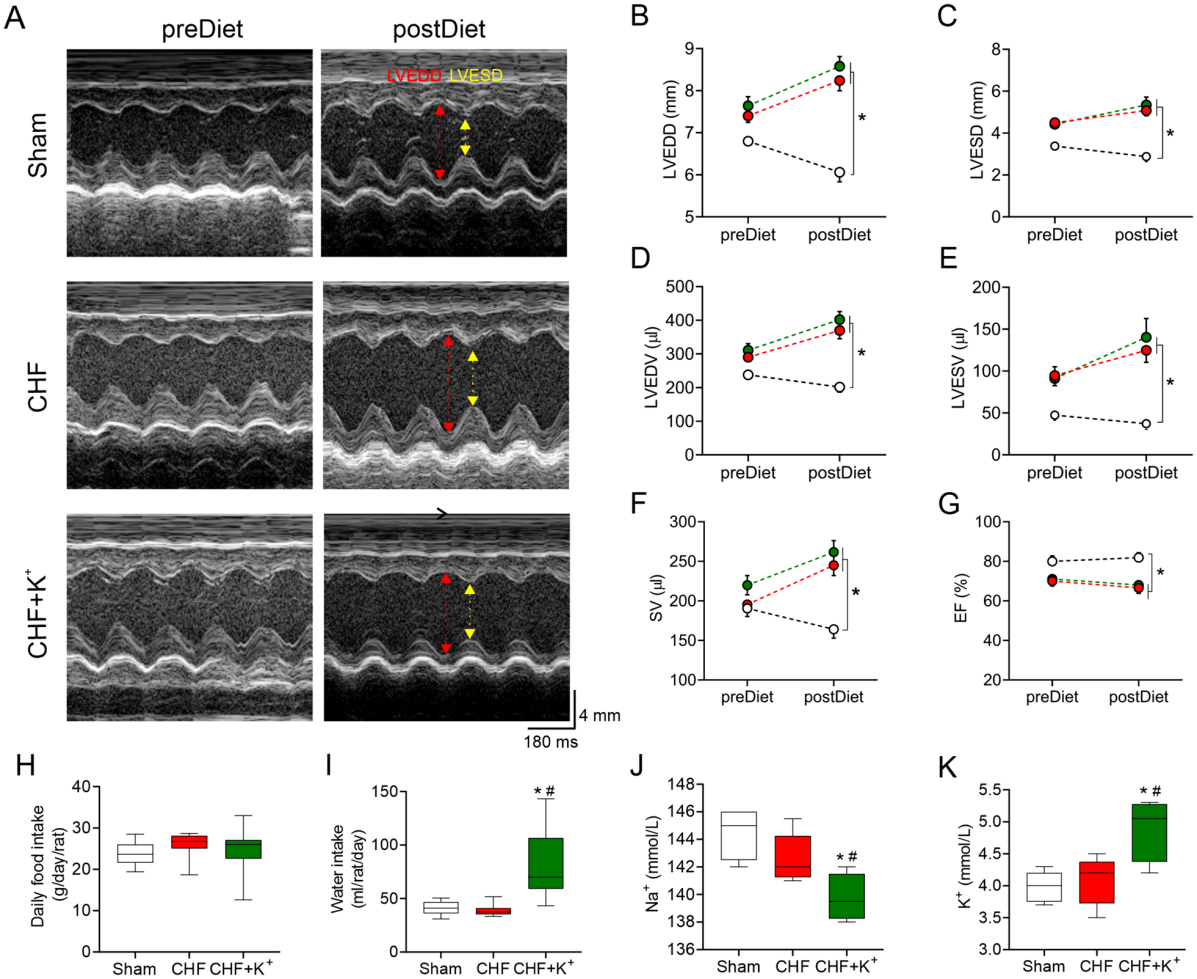
Echocardiographic parameters and plasmatic Na^+^ and K^+^ concentration. **A** Representative echocardiography image of the left ventricle (LV) from one rat per group. LV-end systolic diameter (LVESD, yellow arrow) and LV-end diastolic diameter (LVEDD, red arrow). **B** LVEDD. **C** LVESD. **D** LV-end diastolic volume (LVEDV). **E** LV-end systolic volume (LVESV) (**F**) Stroke volume (SV). **G** Ejection fraction (EF). Note that K^+^ dietary supplementation has no effects on cardiac diameters and volumes in CHF condition. (**H**) Daily food (g/day/rat) and **I** water intake (ml/rat/day) in Sham, CHF and CHF+K^+^ groups. **J** Summary data showing sodium (Na^+^) and **K** potassium (K^+^) ion concentration (mmol/L) in all groups. Note that Na^+^ concentration decreases in CHF+K^+^ while K^+^ concentration is significantly higher. *p < 0.05 vs Sham, ^#^p < 0.05 vs CHF.Holm Sidak post hoc after One-Way ANOVA, n = 5 rats

Both CHF and CHF+K^+^ had a significant increase in LVEDV and LVESV compared to Sham rats (Fig. 5). Furthermore, CHF rats under K^+^ supplemented diet showed similar stroke volume, LV chamber diameter and ejection fraction compared to untreated CHF rats (Fig. 5A-G; Additional file 1: Table S5). Accordingly, cardiac hypertrophy index was not different in CHF animals compared to CHF+K^+^ animals (Additional file 1: Table S5). No difference in body weight (BW) at the end of the experimental protocol were found between experimental conditions (Additional file 1: Table S5). Indeed, daily food intake was similar between groups (Fig. 5H). Plasma levels of [Na^+^] and [K^+^] were comparable between CHF animals and Sham animals (Fig. 5J, K). On the contrary, dietary K^+^ supplementation in CHF significantly increased plasma [K^+^] concentrations and reduced [Na^+^] concentration when compared to CHF untreated animals (Fig. 5J, K). Accordingly, [Na^+^/K^+^] plasma ratio was significantly lower in CHF+K^+^ (28.8 ± 1.2 vs. 35.2 ± 1.6, CHF+K^+^ vs. CHF, respectively; p < 0.05).

### Effects of dietary K^+^ supplementation on cardiac function in CHF

**Fig. 6:**
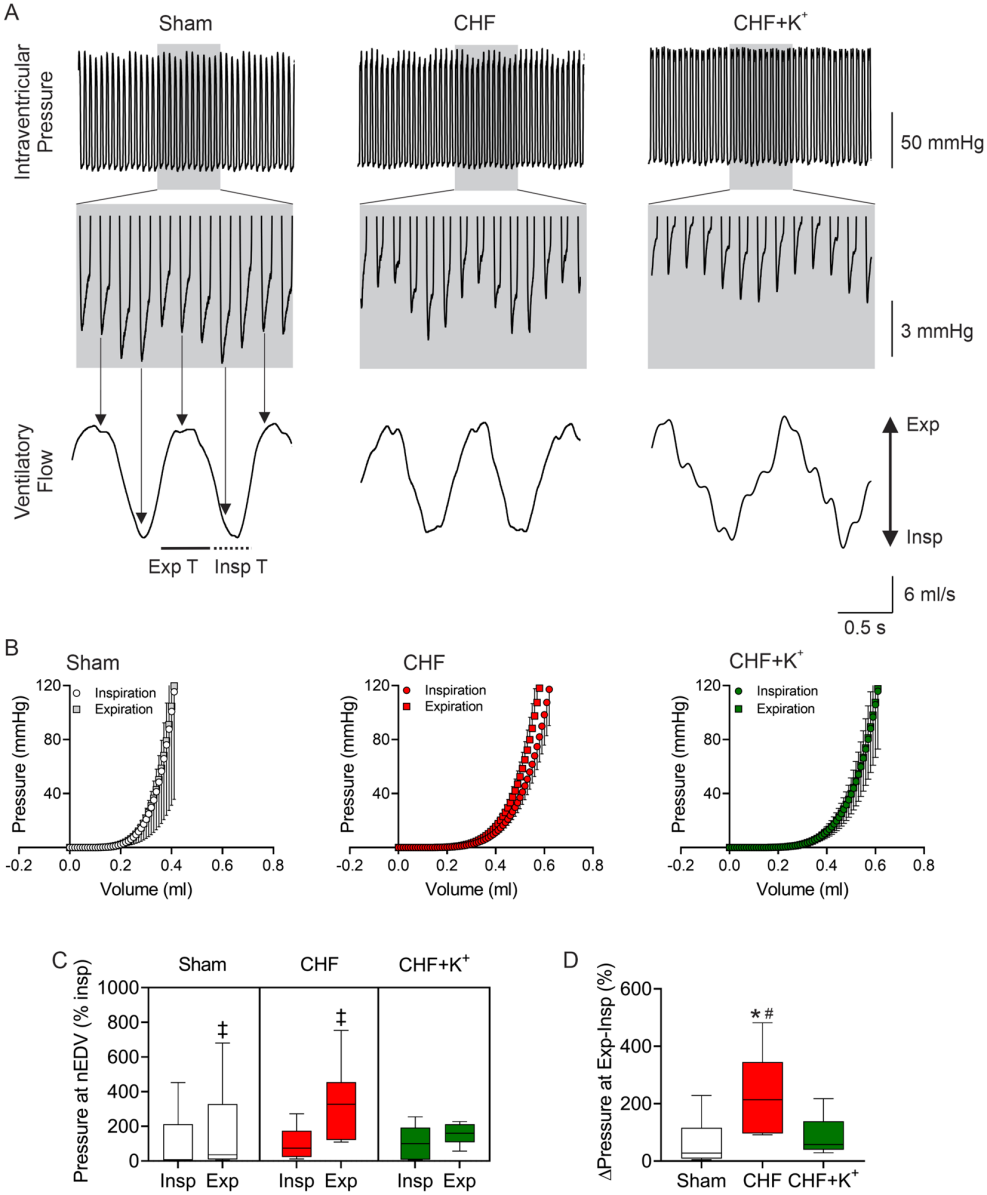
Dietary K^+^ supplementation improves cardiac diastolic function in CHF rats. **A** Representative recording of left ventricle (LV) intraventricular pressure from one Sham rat, one CHF rat and one CHF+K^+^ rat (Upper panel). Lower panel shows ventilatory flows in each section. Note that end diastolic pressure (EDP) is modulated by the ventilatory cycle. **B** End diastolic pressure volume relationship assessed by single-beat PV-loop analysis during the expiratory and inspiratory phases of the breathing cycle. **C** Summary data of normalized EDP (nEDP) during inspiration and expiration. Note that the EDP was severely modulated by the ventilatory cycle in CHF rats and this was abolished by K^+^ diet supplementation. **D** Summary data showing percent changes in Δintraventricular pressures at Exp-Insp. Two-way ANOVA (**C**) and One-way ANOVA (**D**), followed by Holm Sidak *posthoc*. ^‡^P < 0.05 vs. Insp; *P < 0.05 vs. Sham, #P < 0.05 vs CHF+K^+^ n = 5 rats per group

Cardiac diastolic dysfunction has been described in experimental non-ischemic CHF [16, 33]. Indeed, we found a significant increase in LV-end diastolic pressure (LVEDP) in CHF rats compared to Sham rats and this was not significantly reduced by enriched-K^+^ diet in CHF (Additional file 1: Table S6). Since cardiac diastolic function is influenced by ventilation, we determined ventilation-dependent modulation of cardiac function during each inspiratory and expiratory cycles [40]. Intraventricular pressures at the end of diastole (nEDV) during inspiration (ins) were similar between Sham and CHF groups (Fig. 6A, B). However, CHF animals showed a ~ twofold increase in intraventricular pressures during expiration (exp) (332.9 ± 95.0 vs. 162.8 ± 108.2%exp, CHF vs. Sham, respectively; p < 0.05). In CHF+K^+^ animals, the exacerbated increase in nEDV during expiration was abolished and the values were comparable to ones obtained in Sham rats (159.9 ± 28.7 vs. 162.8 ± 108.2%exp, CHF+K^+^ vs. Sham, respectively; p < 0.05). Accordingly, ΔPressures between exp-ins were larger in CHF rats compared to Sham rats (232.9 ± 59.6 vs. 67.8 ± 34.9%, CHF vs. Sham, respectively; p < 0.05) and this effect was absent in CHF+K^+^ rats (Fig. 6C, D).

## Discussion

This proof-of-concept study describes, for the first time, the beneficial effects of daily dietary K^+^ supplementation on major pathophysiological (i.e. autonomic imbalance, breathing disorders) mechanisms associated with the development/maintenance/progression of non-ischemic CHF. We found that dietary K^+^ supplementation improved: (i) cardiac autonomic control balance; (ii) arrhythmia incidence; (iii) breathing pattern regularity; (iv) central respiratory chemoreflex; and (v) cardiorespiratory coupling. Together, our results support that K^+^ supplementation of the diet improves cardiorespiratory outcomes in CHF.

Increasing dietary K^+^ levels has been linked to several cardiovascular benefits. Indeed, evidence from animal studies, clinical trials and meta-analyses have shown that enriched potassium diets are closely linked to marked reductions in the incidence of stroke, protects against vascular injury and reduces cardiac oxidative damage [24, 26, 41–45]. Here, we showed that supplementation of an enriched K^+^ diet in experimental non-ischemic CHF markedly reduces sympathoexcitation, normalizes chemoreflex function and improves cardiac function. The precise mechanism underlying the salutary benefits of enriched-K^+^ diets in cardiac failure are not known. However, decreases in cardiac pre-load has been associated to daily ingestion of high K^+^ diets. Indeed, dietary K^+^ supplementation decreases the expression of the Na^+^-Cl^−^ contransporter (NCC) in the distal nephron which reduce Na^+^ circulating levels (less reabsortion), and leads to effective reductions in circulating blood volume [45–47]. Our results showed that enriched K^+^ diet in CHF significantly decreased plasma [Na^+^]. The later support that high K^+^ diet reduced Na^+^ reabsortion in CHF and suggests that high K^+^ may also reduce circulating volume being the outcome an improved cardiac function due to volume unloading. Further studies will be needed to totally address whether enriched K^+^ diets in CHF elicit changes in circulating blood volume.

It has been shown that during early stage of CHF, sympathoexcitation and RAS activation act as an adaptative physiological mechanism to improve cardiovascular function; however, eventually it becomes maladaptive and sustain disease progression [15, 48]. Importantly, chronic sympathetic hyperactivity and RAS promotes arrhythmia incidence and the further deterioration of cardiac function [7, 27, 33]. One major neuronal network that has been proposed to contribute, at least in part, to sustained high sympathetic activity in CHF encompassed the subfornical organ, paraventricular nucleus of the hypothalamus and the rostral ventrolateral medulla (SFO-PVN-RVLM) [15]. SFO is a highly vascularized nuclei with a permeable blood brain barrier (BBB) and is considered a major source of brain AngII [15, 49, 50]. Furthermore, it has been shown that AngII elicits increase in sympathetic outflow by acting on the SFO/PVN [6, 15, 51] or directly on the RVLM once BBB is disrupted [52]. The specific mechanisms underlying AngII-induced sympathoexcitation in the setting of CHF have not been fully dilucidated. However, one accepted mechanism is associated with AngII-induced oxidative stress and neuronal hyperexcitability [15, 53]. Indeed, activation of AT_1_R which is present in the SFO, PVN and RVLM promotes ROS formation via NADPH oxidase [15, 50, 53–55]. Accordingly, experimental non-ischemic CHF rats showed increased levels of phosphorylated NADPH oxidase in the RVLM and this was closely linked to ROS levels and autonomic imbalance [17]. In the present study we found that dietary K^+^ supplementation in CHF rats reduced the heightened cardiac sympathetic outflow and reduced arrhythmogenesis. It is worth noting that the present study was not intended to provide a detailed description of the molecular signaling pathways by which enriched K^+^ diets reduced sympathoexcitation in CHF but rather to highlight its potential to improve cardiovascular and ventilatory outcomes. However, previous studies showing that dietary K^+^ downregulates renin and angiotensin-I converting enzyme and reduce AngII generation [22, 23] allow us to speculate that one potential mechanism associated with the beneficial effects of K^+^ supplementation in CHF may be linked to RAS regulation. Future studies should focus on uncovering the molecular footprints related to K^+^ supplementation and cardiovascular regulation in the setting of CHF.

Altered chemoreflex function play a pivotal role in the pathophysiology of CHF [16, 31, 56–58]. Indeed, an increased ventilatory response to hypercapnia associated with an enhanced chemoreceptor function is associated with heightened sympathetic nerve activity, cardiac arrhythmogenesis and breathing disturbances in humans [56, 59]. More importantly, the former is closely linked to higher mortality risk [59]. Here, we confirmed and extend previous results showing that in CHF alterations in chemoreflex response are associated with cardiorespiratory disorders [16, 31, 58], but also added new and novel findings showing that dietary K^+^ supplementation represents a useful strategy to normalize chemoreflex function in CHF. How enriched K^+^ diet reduced central chemoreflex sensitivity in non-ischemic CHF is unknown and deserves future investigations. Interestingly, data from epidemiological studies strongly suggests that healthy dietary interventions (i.e. K^+^ supplemented diets) may improve cardiovascular outcomes in several diseases [19–21]. Here, we provided the first evidence showing that daily dietary K^+^ supplementation significantly improves both cardiovascular and respiratory function in experimental non-ischemic CHF. Future and larger studies are needed to fully uncover the salutary potential of dietary K^+^ supplementation in CHF pathophysiology.

Several limitations are inherent in our study. Experimental CHF shows several relevant pathophysiological hallmarks of human non-ischemic CHF such as autonomic imbalance, increased cardiac arrhythmogenesis, alterations in breathing and cardiorespiratory coupling. However, it lacks cardiometabolic disorders (i.e. diabetes mellitus, hypertension, obesity) which has been associated with non-ischemic CHF onset and progression. Nevertheless, we found that K^+^ diet supplementation markedly improved cardiorespiratory outcomes in experimental non-ischemic CHF. Though, caution should be taken when extrapolating these results into human non-ischemic CHF due to the well-known differences between rodents and large mammal physiology. Also, we cannot rule out long-term effects of K^+^ supplemented diets on CHF. However, Chang and cols. [60] showed that adult subjects receiving potassium-enriched diets for 31 months displayed significantly lower cardiovascular disease mortality. Also, potassium-enriched diets appear to be well tolerated up to 31 months in humans. Whether this can be directly translated into human non-ischemic CHF patients is not clear. Future studies in both experimental and human non-ischemic CHF are required to fully determine the effects of long-term administration of potassium-enriched diets on cardiorespiratory function.

## Conclusion

Together, dietary K^+^ supplementation exerts beneficial effects on cardiorespiratory function in experimental CHF. This study provide first comprehensive physiological data that support the salutary effects of K^+^ supplementation on the maintenance of heightened cardiac sympathetic activity and breathing disturbances in CHF. In addition, we acknowledge that this study was not intended to provide the exact cellular mechanism of dietary K^+^- mediated physiological outcomes. Accordingly, upcoming research should focus on the cellular/molecular mechanisms associated with the beneficial effects of daily dietary K^+^ supplementation in the setting of non-ischemic CHF.

## Supplementary Information

**Additional file 1: Table S1.** Experimental diets compositions. **Table S2.** Effect of K^+^ supplementation on respiratory disorders incidence in CHF condition. **Table S3.** Effects of K^+^ supplemented in the diet on ventilatory parameters in CHF rats. **Table S4.** Arterial blood pressure and dietary K^+^ supplementation in CHF. **Table S5.** Echocardiography parameters. **Table S6.** Effect of K^+^ supplemented diet on intraventricular cardiac parameters.

## Data Availability

The data presented in this study are available on request from the corresponding author. The data are not publicly available due to current funding source restrictions.

## Abbreviations

AngII: Angiotensin II
AT_1_R: AngII receptor type I
BRS: Baroreflex sensitivity
BW: Body weight
CHF: Chronic heart failure
DBP: Diastolic blood pressure
EF: Ejection fraction
Exp: Expiratory phase
FS: Fractional shortening
HCVR: Hypercapnic ventilatory response
HR: Heart rate
HVR: Hypoxic ventilatory response
HW: Heart weight
Ins: Inspiratory phase
IS: Irregularity sore
K^+^: Potassium
LVEDD: Left ventricular end diastolic diameter
LVEDP: Left ventricular end diastolic pressure
LVEDV: Left ventricular end diastolic volume
LVESD: Left ventricular end systolic diameter
LVESP: Left ventricular end systolic pressure
LVESV: Left ventricular end systolic volume
MABP: Mean arterial blood pressure
NADPH: Nicotinamide adenine dinucleotide phosphate
nEDP: Normalized end diastolic pressure
PP: Pulse pressure
PV: Pressure–volume
PVN: Paraventricular nucleus
RAS: Renin-angiotensin-system
R_f_: Respiratory frequency
ROS: Reactive oxygen species
RVLM: Rostral ventrolateral medulla
SBP: Systolic blood pressure
SFO: Subfornical organ
SV: Stroke volume
V_E_: Minute volume
V_T_: Tidal volume

## References

[1] Ponikowski P, Anker SD, AlHabib KF, Cowie MR, Force TL, Hu S, Jaarsma T, Krum H, Rastogi V, Rohde LE, Samal UC, Shimokawa H, Siswanto BB, Sliwa K, Filippatos G (2014). Heart failure: preventing disease and death worldwide. Eur J Heart Fail.

[2] Desai AS, Stevenson LW (2012). Rehospitalization for heart failure: predict or prevent?. Circulation.

[3] Yoon S, Eom GH (2019). Heart failure with preserved ejection fraction: present status and future directions. Exp Mol Med.

[4] van der Wal HH, van Deursen VM, van der Meer P, Voors AA (2017). Comorbidities in heart failure. Handb Exp Pharmacol.

[5] Yancy CW, Jessup M, Bozkurt B, Butler J, Casey DE, Colvin MM, Drazner MH, Fonarow GS, Geraci SA, Horwich T, Januzzi JL, Johnson MR, Kasper EK, Levy WC, Masoudi FA, McBride PE, McMurray JJV, Mitchell JE, Peterson PN, Riegel B, Sam F, Stevenson LW, Wilson-Tang WH, Tsai EJ, Wilkoff BL (2013). 2013 ACCF/AHA guideline for the management of heart failure: a report of the American College of Cardiology Foundation/American Heart Association Task Force on Practice Guidelines. J Am Coll Cardiol.

[6] Kishi T (2012). Heart failure as an autonomic nervous system dysfunction. J Cardiol.

[7] Florea VG, Cohn JN (2014). The autonomic nervous system and heart failure. Circ Res.

[8] Franciosi S, Perry FK, Roston TM, Armstrong KR, Claydon VE, Sanatani S (2017). The role of the autonomic nervous system in arrhythmias and sudden cardiac death. Auton Neurosci.

[9] Lanfranchi PA, Braghiroli A, Bosimini E, Mazzuero G, Colombo R, Donner CF, Giannuzzi P (1999). Prognostic value of nocturnal Cheyne-Stokes respiration in chronic heart failure. Circulation.

[10] Bitter T, Westerheide N, Prinz C, Hossain MS, Vogt J, Langer C, Horstkotte D, Oldenburg O (2011). Cheyne-Stokes respiration and obstructive sleep apnoea are independent risk factors for malignant ventricular arrhythmias requiring appropriate cardioverter-defibrillator therapies in patients with congestive heart failure. Eur Heart J.

[11] Sciarretta S, Paneni F, Palano F, Chin D, Tocci G, Rubattu S, Volpe M (2019). Role of the renin-angiotensin-aldosterone system and inflammatory processes in the development and progression of diastolic dysfunction. Clin Sci.

[12] Xu B, Li H (2015). Brain mechanisms of sympathetic activation in heart failure: roles of the renin-angiotensin system, nitric oxide and pro-inflammatory cytokines. Mol Med Rep.

[13] Griendling KK, Minieri CA, Ollerenshaw JD, Alexander RW (1994). Angiotensin II stimulates NADH and NADPH oxidase activity in cultured vascular smooth muscle cells. Circ Res.

[14] Koba S, Hisatome I, Watanabe T (2014). Central command dysfunction in rats with heart failure is mediated by brain oxidative stress and normalized by exercise training. J Physiol.

[15] Díaz HS, Toledo C, Andrade DC, Marcus NJ, Del Rio R (2019). Neuroinflammation in heart failure: new insights for an old disease. J Physiol.

[16] Toledo C, Andrade DC, Lucero C, Arce-Alvarez A, Díaz HS, Aliaga V, Schultz HD, Marcus NJ, Manriquez M, Faúndez M, Del Rio R (2017). Cardiac diastolic and autonomic dysfunction are aggravated by central chemoreflex activation in heart failure with preserved ejection fraction rats. J Physiol.

[17] Andrade DC, Arce-Alvarez A, Toledo C, Díaz HS, Lucero C, Schultz HD, Marcus NJ, Del Rio R (2017). Exercise training improves cardiac autonomic control, cardiac function, and arrhythmogenesis in rats with preserved-ejection fraction heart failure. J Appl Physiol.

[18] Dahl LK, Leitl G, Heine M (1972). Influence of dietary potassium and sodium/potassium molar ratios on the development of salt hypertension. J Exp Med.

[19] Rodrigues SL, Baldo MP, Machado RC, Forechi L, Molina MDCB, Mill JG (2014). High potassium intake blunts the effect of elevated sodium intake on blood pressure levels. J Am Soc Hypertens.

[20] Geleijnse JM, Kok FJ, Grobbee DE (2003). Blood pressure response to changes in sodium and potassium intake: a metaregression analysis of randomised trials. J Hum Hypertens.

[21] Whelton PK, Carey RM, Aronow WS, Casey DE, Collins KJ, Dennison Himmelfarb C, DePalma SM, Gidding S, Jamerson KA, Jones DW, MacLaughlin EJ, Muntner P, Ovbiagele B, Smith SC, Spencer CC, Stafford RS, Taler SJ, Thomas RJ, Williams KA, Williamson JD, Wright JT (2018). 2017 ACC/AHA/AAPA/ABC/ACPM/AGS/APhA/ASH/ASPC/ NMA/PCNA guideline for the prevention, detection, evaluation, and management of high blood pressure in adults: executive summary: a report of the American College of Cardiology/American Heart Association Task Force on Clinical Practice Guidelines. Hypertension.

[22] Gonzalez AA, Gallardo M, Cespedes C, Vio CP (2019). Potassium intake prevents the induction of the renin-angiotensin system and increases medullary ACE2 and COX-2 in the kidneys of angiotensin II-dependent hypertensive rats. Front Pharmacol.

[23] Vio CP, Gallardo P, Cespedes C, Salas D, Diaz-Elizondo J, Mendez N (2020). Dietary potassium downregulates angiotensin-I converting enzyme, renin, and angiotensin converting enzyme 2. Front Pharmacol.

[24] Fujita T, Sato Y (1984). Changes in renal and central noradrenergic activity with potassium in DOCA-salt rats. Am J Physiol.

[25] McCabe RD, Bakarich MA, Srivastava K, Young DB (1994). Potassium inhibits free radical formation. Hypertension.

[26] Matsui H, Shimosawa T, Uetake Y, Wang H, Ogura S, Kaneko T, Liu J, Ando K, Fujita T (2006). Protective effect of potassium against the hypertensive cardiac dysfunction: association with reactive oxygen species reduction. Hypertension.

[27] Zucker IH, Xiao L, Haack KK (2014). The central renin–angiotensin system and sympathetic nerve activity in chronic heart failure. Clin Sci.

[28] Weaver CM (2013). Potassium and health. Adv Nutr.

[29] World Health Organization (2012). Guideline: potassium intake for adults and children.

[30] Bowling CB, Pitt B, Ahmed MI, Aban IB, Sanders PW, Mujib M, Campbell RC, Love TE, Aronow WS, Allman RM, Bakris GL, Ahmed A (2010). Hypokalemia and outcomes in patients with chronic heart failure and chronic kidney disease: findings from propensity-matched studies. Cir Heart Fail.

[31] Diaz HS, Andrade DC, Toledo C, Pereyra KV, Schwarz KG, Díaz-Jara E, Lucero C, Arce-Alvarez A, Schultz HD, Silva JN, Takakura AC, Moreira TS, Del MNJ, Rio R (2020). Episodic stimulation of central chemoreceptor neurons elicits disordered breathing and autonomic dysfunction in volume overload heart failure. Am J Physiol Lung Cell Mol Physiol.

[32] Abassi Z, Goltsman I, Karram T, Winaver J, Hoffman A (2011). Aortocaval fistula in rat: a unique model of volume-overload congestive heart failure and cardiac hypertrophy. J Biomed Biotechnol..

[33] Andrade DC, Toledo C, Díaz HS, Lucero C, Arce-Álvarez A, Oliveira LM, Takakura AC, Moreira TS, Schultz HD, Marcus NJ, Alcayaga J, Del Rio R (2019). Ablation of brainstem C1 neurons improves cardiac function in volume overload heart failure. Clin Sci.

[34] Toledo C, Andrade DC, Díaz HS, Pereyra KV, Schwarz KG, Díaz-Jara E, Oliveira LM, Takakura AC, Moreira TS, Schultz HD, Marcus N, Del Rio R (2019). Rostral ventrolateral medullary catecholaminergic neurones mediate irregular breathing pattern in volume overload heart failure rats. J Physiol.

[35] Greenwood MP, Greenwod M, Paton JFR, Murphy D (2014). Salt appetite is reduced by a single experience of drinking hypertonic saline in the adult rat. PLoS One..

[36] Rey S, Tarvainen MP, Karjalainen PA, Iturriaga R (2008). Dynamic time-varying analysis of heart rate and blood pressure variability in cats exposed to short-term chronic intermittent hypoxia. Am J Physiol Regul Integr Comp Physiol.

[37] Marcus NJ, Del Rio R, Schultz HD (2014). Central role of carotid body chemoreceptors in disordered breathing and cardiorenal dysfunction in chronic heart failure. Front Physiol.

[38] Oliveira-Sales EB, Toward MA, Campos RR, Paton JF (2014). Revealing the role of the autonomic nervous system in the development and maintenance of Goldblatt hypertension in rats. Auton Neurosci.

[39] Pacher P, Nagayama T, Mukhopadhyay P, Bátkai S, Kass DA (2008). Measurement of cardiac function using pressure-volume conductance catheter technique in mice and rats. Nat Protoc.

[40] Ogilvie LM, Edgett BA, Huber JS, Platt MJ, Eberl H, Lutchmedial S, Brunt KR, Simpson JA (2020). Hemodynamic assessment of diastolic function for experimental models. Am J Physiol Heart Circ Physiol.

[41] Tobian L, Lange J, Ulm K, Wold L, Iwai J (1985). Potassium reduces cerebral hemorrhage and death rate in hypertensive rats, even when blood pressure is not lowered. Hypertension.

[42] Kido M, Ando K, Onozato ML, Tojo A, Yoshikawa M, Ogita T, Fujita T (2008). Protective effect of dietary potassium against vascular injury in salt-sensitive hypertension. Hypertension.

[43] D’Elia L, Barba G, Cappuccio FP, Strazullo P (2011). Potassium intake, stroke, and cardiovascular disease a meta-analysis of prospective studies. J Am Coll Cardiol.

[44] Hunt BD, Cappuccio FP (2014). Potassium intake and stroke risk: a review of the evidence and practical considerations for achieving a minimum target. Stroke.

[45] McDonough AA, Veiras LC, Guevara CA, Ralph DL (2017). Cardiovascular benefits associated with higher dietary K+ vs. lower dietary Na+: evidence from population and mechanistic studies. Am J Physiol Endocrinol Metab..

[46] Kortenoeven MLA, Esteva-Font C, Dimke H, Poulsen SB, Murali SK, Fenton RA (2021). High dietary potassium causes ubiquitin-dependent degradation of the kidney sodium-chloride cotransporter. J Biol Chem..

[47] Veiras LC, Han J, Ralph DL, McDonough AA (2016). Potassium supplementation prevents sodium chloride cotransporter stimulation during angiotensin II hypertension. Hypertension.

[48] Triposkiadis F, Karayannis G, Giamouzis G, Skoularigis J, Louridas G, Butler J (2009). The sympathetic nervous system in heart failure physiology, pathophysiology, and clinical implications. J Am Coll Cardiol.

[49] Llewellyn TL, Sharma NM, Zheng H, Patel KP (2014). Effects of exercise training on SFO-mediated sympathoexcitation during chronic heart failure. Am J Physiol Heart Circ Physiol.

[50] Wang HW, Huang BS, White RA, Chen A, Ahmad M, Leenen FH (2016). Mineralocorticoid and angiotensin II type 1 receptors in the subfornical organ mediate angiotensin II–induced hypothalamic reactive oxygen species and hypertension. Neuroscience.

[51] Yoshimura R, Sato T, Kawada T, Shishido T, Inagaki M, Miyano H, Nakahara T, Mitashita H, Takaki H, Tatewaki T, Yanagiya Y, Sugimachi M, Sunagawa K (2000). Increased brain angiotensin receptor in rats with chronic high-output heart failure. J Card Fail.

[52] Biancardi VC, Stern JE (2016). Compromised blood–brain barrier permeability: novel mechanism by which circulating angiotensin II signals to sympathoexcitatory centres during hypertension. J Physiol.

[53] Campese VM, Shaohua YE, Huiquin Z (2005). Oxidative stress mediates angiotensin II–dependent stimulation of sympathetic nerve activity. Hypertension.

[54] Hanna IR, Taniyama Y, Szöcs K, Rocic P, Griendling KK (2002). NAD(P)H oxidase-derived reactive oxygen species as mediators of angiotensin II signaling. Antioxid Redox Signal.

[55] Chan SHH, Hsu KS, Huang CC, Wang LL, Ou CC, Chan JYH (2005). NADPH oxidase–derived superoxide anion mediates angiotensin ii–induced pressor effect via activation of p38 mitogen–activated protein kinase in the rostral ventrolateral medulla. Circ Res.

[56] Giannoni A, Emdin M, Poletti R, Bramanti F, Prontera C, Piepoli M, Passino C (2008). Clinical significance of chemosensitivity in chronic heart failure: influence on neurohormonal derangement, Cheyne-Stokes respiration and arrhythmias. Clin Sci.

[57] Giannoni A, Emdin M, Bramanti F, Iudice G, Francis DP, Barsotti A, Piepolo M, Passino C (2009). Combined increased chemosensitivity to hypoxia and hypercapnia as a prognosticator in heart failure. J Am Coll Cardiol.

[58] Yamada K, Asanoi H, Ueno H, Joho S, Takagawa J, Kameyama T, Hirai T, Nozawa T, Inoue H (2004). Role of central sympathoexcitation in enhanced hypercapnic chemosensitivity in patients with heart failure. Am Heart J.

[59] Brack T, Thüer I, Clarenbach CF, Senn O, Noll G, Russi EW, Bloch KE (2007). Daytime Cheyne-Stokes respiration in ambulatory patients with severe congestive heart failure is associated with increased mortality. Chest.

[60] Chang HY, Hu YW, Yue CSJ, Wen YW, Yeh WT, Hsu LS, Tsai SY, Pan WH (2006). Effect of potassium-enriched salt on cardiovascular mortality and medical expenses of elderly men. Am J Clin Nutr.

