# Correction to: Effects of enriched-potassium diet on cardiorespiratory outcomes in experimental non-ischemic chronic heart failure

**DOI:** 10.1186/s40659-022-00370-w

**Published:** 2022-01-21

**Authors:** Karla G. Schwarz, Katherin V. Pereyra, Camilo Toledo, David C. Andrade, Hugo S. Díaz, Esteban Díaz-Jara, Domiziana Ortolani, Angélica Rios-Gallardo, Paulina Arias, Alexandra Las Heras, Ignacio Vera, Fernando C. Ortiz, Nibaldo C. Inestrosa, Carlos P. Vio, Rodrigo Del Rio

**Affiliations:** 1 Laboratory of Cardiorespiratory Control, Department of Physiology, Pontificia Universidad Católica de Chile, Santiago, Chile; 2 Centro de Excelencia en Biomedicina de Magallanes (CEBIMA), Universidad de Magallanes, Punta Arenas, Chile; 3 Centro de Fisiología y Medicina de Altura, Departamento Biomedico, Facultad de Ciencias de la Salud, Universidad de Antofagasta, Antofagasta, Chile; 4 Mechanisms of Myelin Formation and Repair Laboratory, Instituto de Ciencias Biomédicas, Facultad de Ciencias de Salud, Universidad Autónoma de Chile, Santiago, Chile; 5 Facultad de Medicina y Ciencia, Universidad San Sebastián, Santiago, Chile; 6 Centro de Envejecimiento y Regeneración (CARE), Pontificia Universidad Católica de Chile, Santiago, Chile

## Correction to: Biological Research (2021) 54:43 https://doi.org/10.1186/s40659-021-00365-z

Following publication of the original article [1], the Figs. 2, 3 and 4 are misplaced. The correct order of figures is given in this erratum (Figs. 1, 2, 3, 4, 5, 6).

**Fig. 1:**
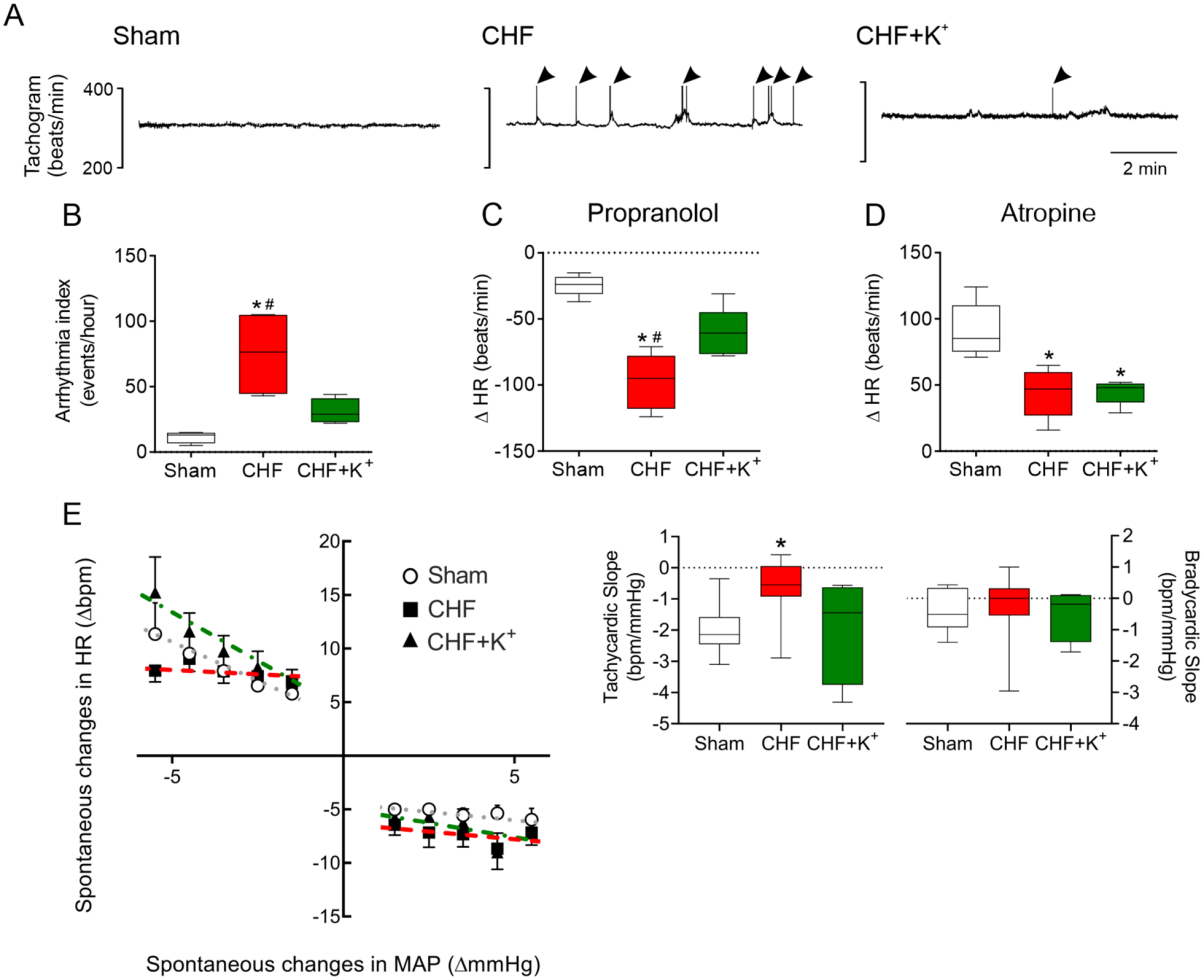
Dietary K^+^ supplementation decreases arrhythmia incidence, cardiac sympathetic tone and improves spontaneous baroreflex in CHF rats. **A** Representative recording of heart rate (HR) tachograms obtained from one Sham rat, one CHF rat and one CHF+K^+^ rat. Arrowheads indicate arrhythmic events. Note that K^+^ supplemented diet reduces arrhythmic events in CHF. **B** Summary data showing arrhythmia index (events/hour). **C** Heart rate responses (ΔHR) after sympathetic blockade with propranolol (1 mg/kg). **D** ΔHR after parasympathetic blockade with atropine (1 mg/kg). **E** Baroreflex sensitivity (BRS) during spontaneous changes in HR and mean arterial pressure (MAP). Each dashed line represents the tachycardic or bradycardic slope. *P < 0.05 vs Sham, ^#^P < 0.05 vs CHF+K^+^. Holm Sidak post hoc after One-Way ANOVA, n = 5 rats per group

**Fig. 2:**
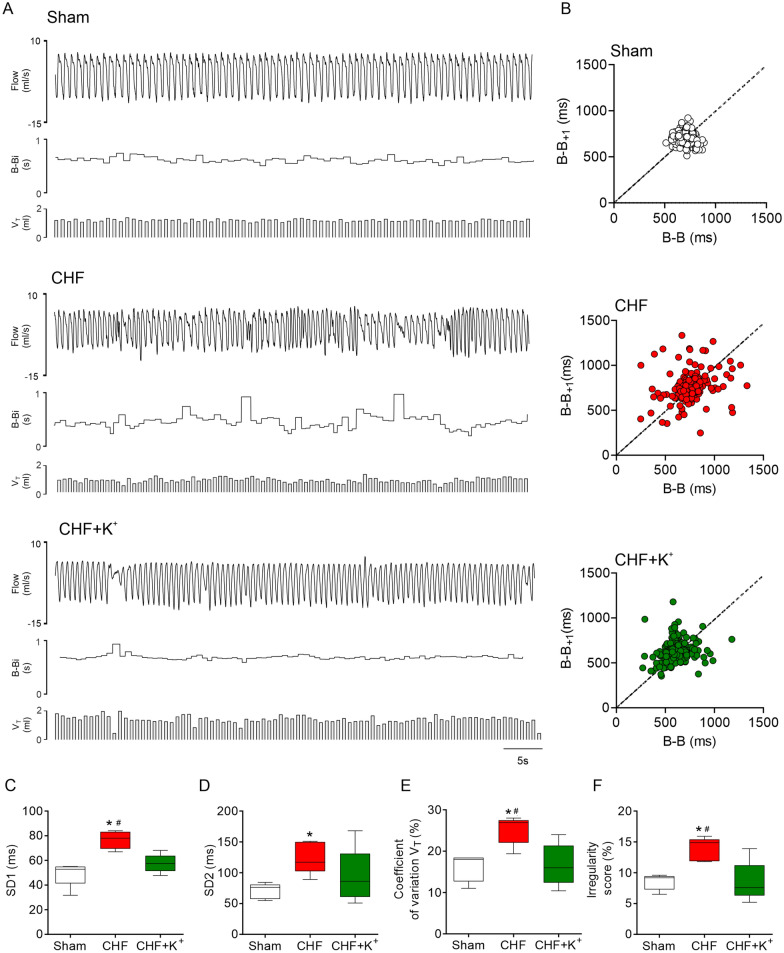
Daily dietary K^+^ supplementation improves breathing in CHF rats. **A** Representative ventilation recordings of ventilatory flow (ml/s), breath-to-breath interval (B–B_i_, s) and tidal volume (V_T_, ml) obtained from one Sham rat, one CHF rat and one CHF+K^+^ rat. **B** Representative Poincare plots showing B–B_i_ variability. **C**–**D** Summary data displaying SD1 and SD2 in all groups. Note that irregularity of B–B_i_ in CHF is markedly improve by dietary K^+^ supplementation. **E** Summary data showing changes in breathing irregularity score (%). **F** Coefficient of variation of V_T_ amplitudes (%). K^+^ supplemented diet significantly reduces V_T_ oscillations in CHF. *P < 0.05 vs Sham, ^#^P < 0.05 vs CHF+K^+^. Holm Sidak post hoc after One-Way ANOVA, n = 5 rats per group

**Fig. 3:**
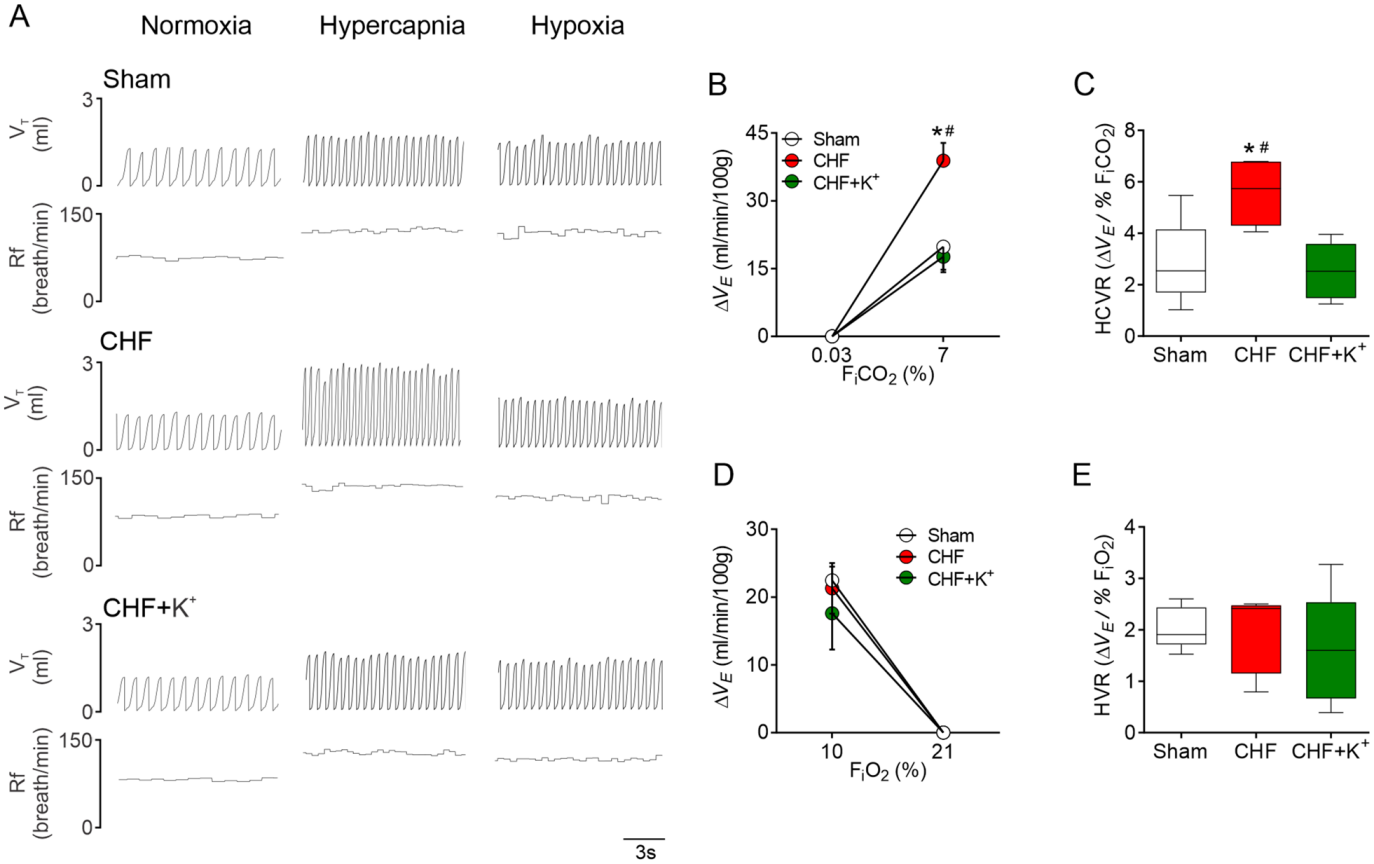
Central chemoreflex drive is normalized by K^+^ supplementation in CHF rats. **A** Representative recording of tidal volume (V_T_) and respiratory frequency (Rf) during normoxia (F_i_O_2_ 21%), hypercapnia (F_i_CO_2_ 7%) and hypoxia (F_i_O_2_ 10% in one Sham rat, one CHF rat and one CHF+K^+^ rat. **B**–**C** Summary data showing the magnitude (ΔV_E_, ml/min/100 g) and gain (ΔV_E_/%F_i_CO_2_) of the ventilatory response to hypercapnia (HCVR). Note that K^+^ supplementation totally restored normal HCVR in CHF rats. **D**–**E** Summary data showing the magnitude (ΔV_E_, ml/min/100 g) and gain (ΔV_E_/%F_i_O_2_) of the hypoxic ventilatory response (HVR). *P < 0.05 vs Sham, ^#^P < 0.05 vs CHF+K^+^. Holm Sidak post hoc after One-Way ANOVA, n = 5 rats per group

**Fig. 4:**
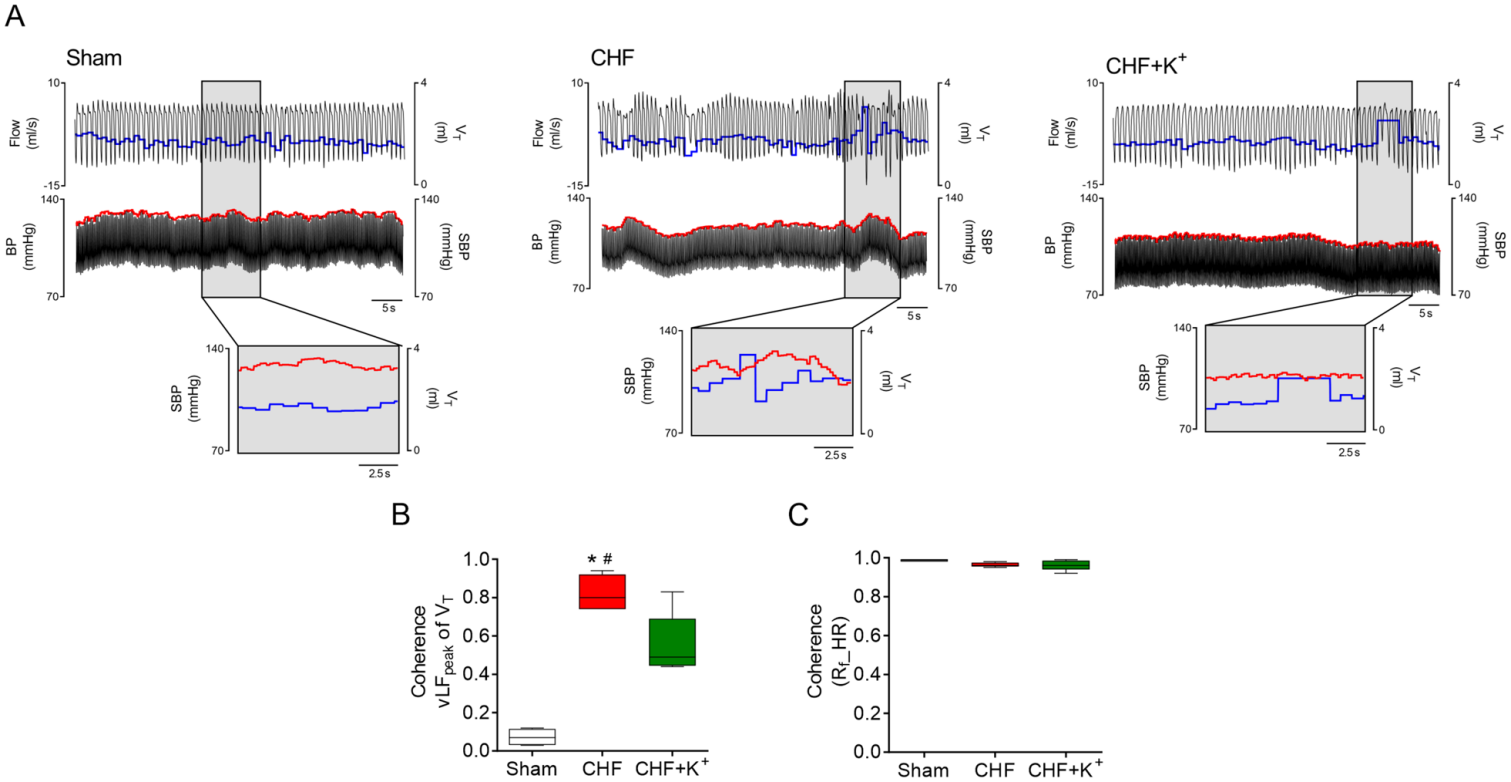
Dietary K^+^ supplementation attenuates cardiorespiratory coupling in CHF. **A** Representative traces of ventilatory flow (Flow, ml/s) and arterial blood pressure (BP, mmHg) in one Sham rat, one CHF rat and one CHF+K^+^ rat. Tidal volume (V_T_) is marked in blue while systolic blood pressure (SBP, mmHg) is shown in red. Note that in CHF rats, oscillations in ventilation are phase with increases in SBP, reflecting a positive interaction between signals. **B** Summary data showing the magnitude of coherence between V_T_ and SBP centered at the very low frequency (vLF) peak of V_T_. **C** Summary data showing coherence function between respiratory frequency (R_F_) and heart rate (HR). *P < 0.05 vs Sham, ^#^P < 0.05 vs CHF+K^+^. Holm Sidak post hoc after One-Way ANOVA, n = 5 rats per group

**Fig. 5:**
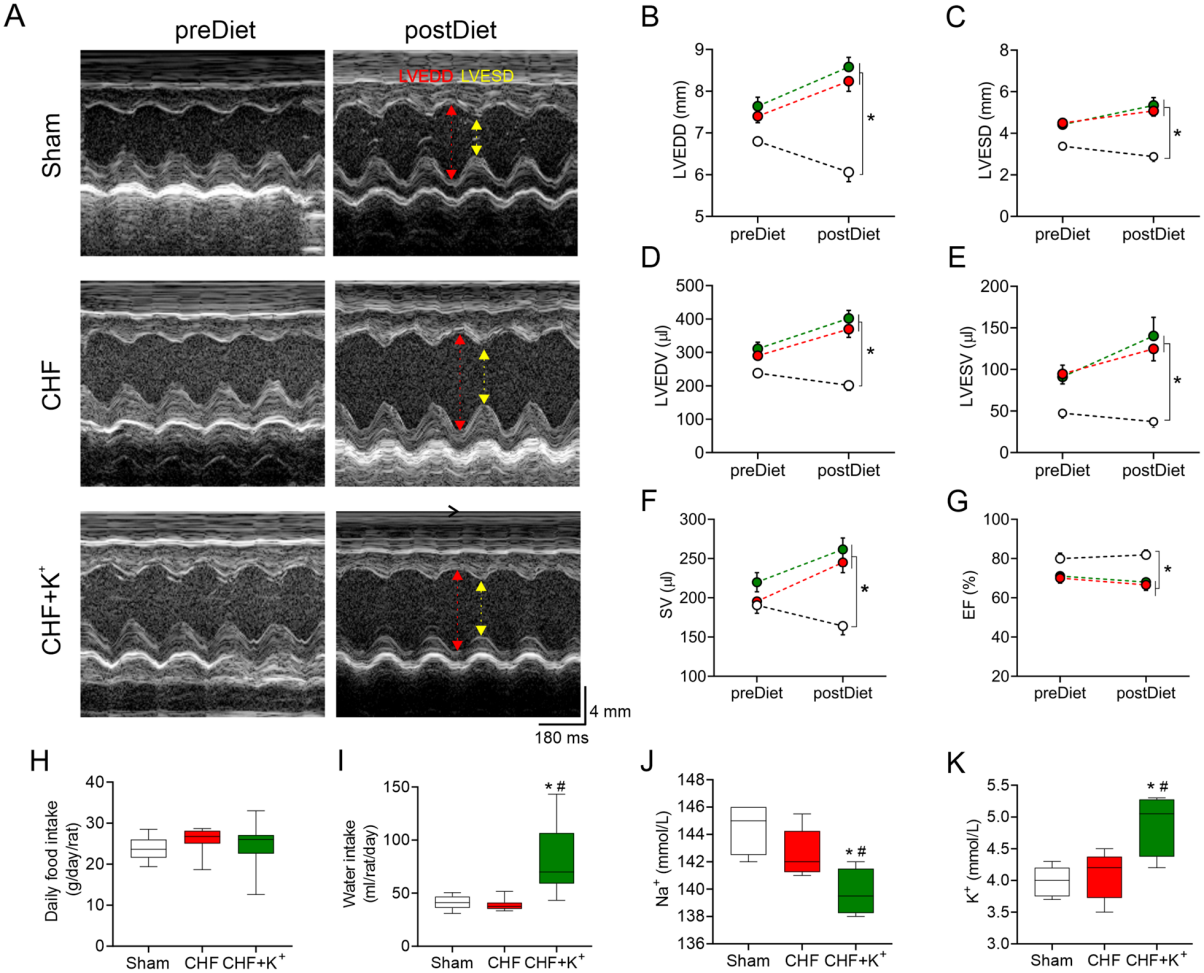
Echocardiographic parameters and plasmatic Na^+^ and K^+^ concentration. **A** Representative echocardiography image of the left ventricle (LV) from one rat per group. LV-end systolic diameter (LVESD, yellow arrow) and LV-end diastolic diameter (LVEDD, red arrow). **B** LVEDD. **C** LVESD. **D** LV-end diastolic volume (LVEDV). **E** LV-end systolic volume (LVESV) (**F**) Stroke volume (SV). **G** Ejection fraction (EF). Note that K^+^ dietary supplementation has no effects on cardiac diameters and volumes in CHF condition. (**H**) Daily food (g/day/rat) and **I** water intake (ml/rat/day) in Sham, CHF and CHF+K^+^ groups. **J** Summary data showing sodium (Na^+^) and **K** potassium (K^+^) ion concentration (mmol/L) in all groups. Note that Na^+^ concentration decreases in CHF+K^+^ while K^+^ concentration is significantly higher. *p < 0.05 vs Sham, ^#^p < 0.05 vs CHF.Holm Sidak post hoc after One-Way ANOVA, n = 5 rats

**Fig. 6:**
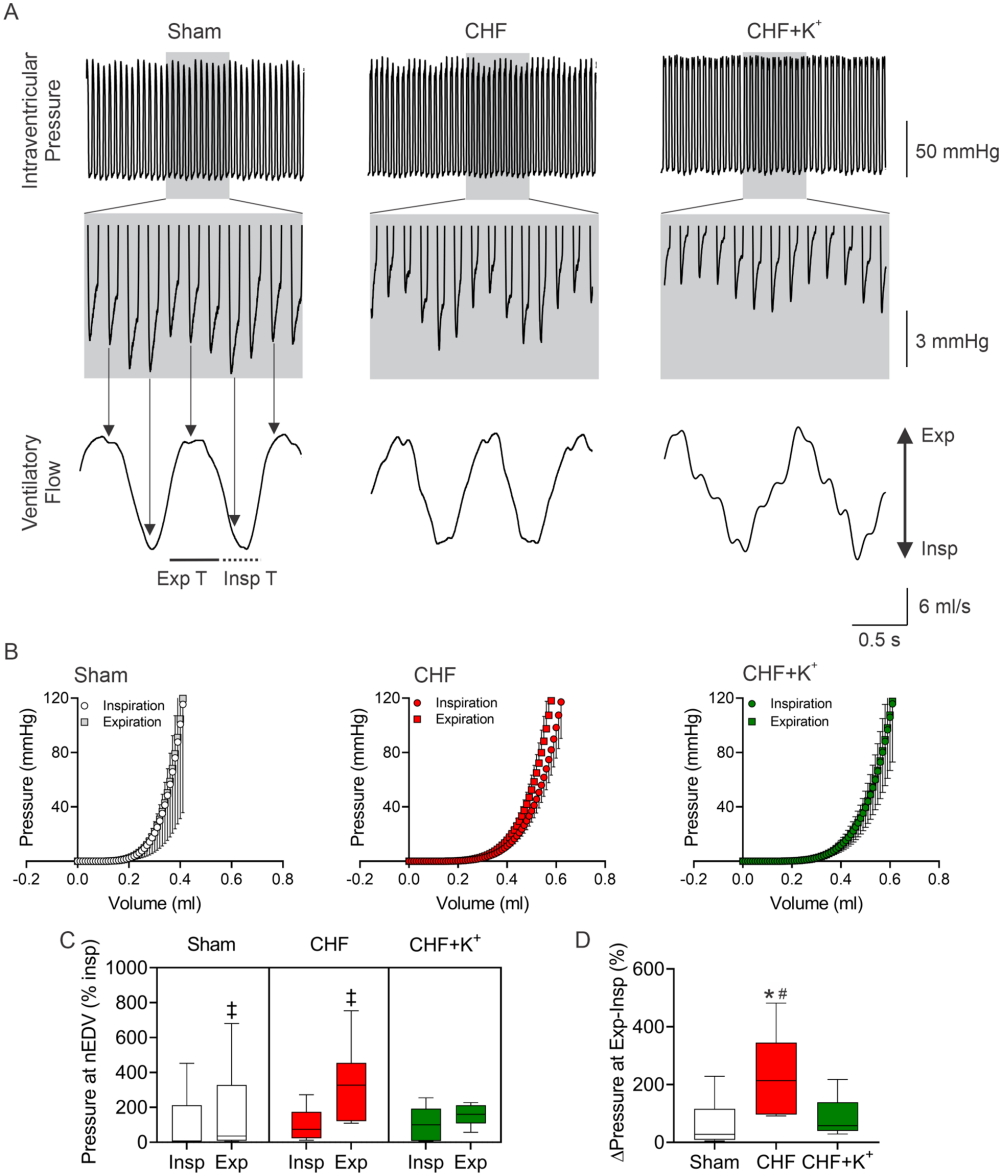
Dietary K^+^ supplementation improves cardiac diastolic function in CHF rats. **A** Representative recording of left ventricle (LV) intraventricular pressure from one Sham rat, one CHF rat and one CHF+K^+^ rat (Upper panel). Lower panel shows ventilatory flows in each section. Note that end diastolic pressure (EDP) is modulated by the ventilatory cycle. **B** End diastolic pressure volume relationship assessed by single-beat PV-loop analysis during the expiratory and inspiratory phases of the breathing cycle. **C** Summary data of normalized EDP (nEDP) during inspiration and expiration. Note that the EDP was severely modulated by the ventilatory cycle in CHF rats and this was abolished by K^+^ diet supplementation. **D** Summary data showing percent changes in Δintraventricular pressures at Exp-Insp. Two-way ANOVA (**C**) and One-way ANOVA (**D**), followed by Holm Sidak *posthoc*. ^‡^P < 0.05 vs. Insp; *P < 0.05 vs. Sham, #P < 0.05 vs CHF+K^+^ n = 5 rats per group

The original article has been corrected.
