# Supplementary material for: Effects of enriched-potassium diet on cardiorespiratory outcomes in experimental non-ischemic chronic heart failure
Source: Biol Res. 2021 Dec 24;54:43. doi: 10.1186/s40659-021-00365-z (PMC8710008; doi:10.1186/s40659-021-00365-z)
Supplement: Supplementary file 1 — Additional file 1: Table S1. Experimental diets compositions. Table S2. Effect of K+ supplementation on respiratory disorders incidence in CHF condition. Table S3. Effects of K+ supplemented in the diet on ventilatory parameters in CHF rats. Table S4. Arterial blood pressure and dietary K+ supplementation in CHF. Table S5. Echocardiography parameters. Table S6. Effect of K+ supplemented diet on intraventricular cardiac parameters. [file 40659_2021_365_MOESM1_ESM.pdf]

Supplemental Information

Effects of Enriched-Potassium Diet on Cardiorespiratory Outcomes in Experimental Non-ischemic Chronic Heart Failure

Karla G. Schwarz [1], Katherin V. Pereyra [1], Camilo Toledo [1,2], David C. Andrade [1,3], Hugo S. Díaz [1], Esteban Díaz-Jara [1], Domiziana Ortolani [1], Angélica Rios [1,2], Paulina Arias [1], Alexandra Las Heras [1], Ignacio Vera [1], Fernando C. Ortiz [4], Nibaldo C. Inestrosa [2,6], Carlos P. Vio [5,6] , Rodrigo Del Rio [1,2,6*]

[1]Laboratory of Cardiorespiratory Control, Department of Physiology, Pontificia Universidad Católica de Chile, Santiago, Chile. [2]Centro de Excelencia en Biomedicina de Magallanes (CEBIMA), Universidad de Magallanes, Punta Arenas, Chile. [3]Centro de Fisiología y Medicina de Altura, Departamento Biomedico, Facultad de Ciencias de la Salud, Universidad de Antofagasta, Antofagasta, Chile. [4]Institute of Biomedical Sciences, Universidad Autónoma de Chile, Santiago. [5]Facultad de Medicina y Ciencia, Universidad San Sebastián, Santiago, Chile. [6]Centro de Envejecimiento y Regeneración (CARE), Pontificia Universidad Católica de Chile, Santiago, Chile.

***Corresponding author:** Rodrigo Del Rio, Ph.D.
Laboratory of Cardiorespiratory Control
Pontificia Universidad Católica de Chile.
Santiago, Chile.
Tel: +562 23542859
E-mail: rdelrio@bio.puc.cl

Supplemental Figure

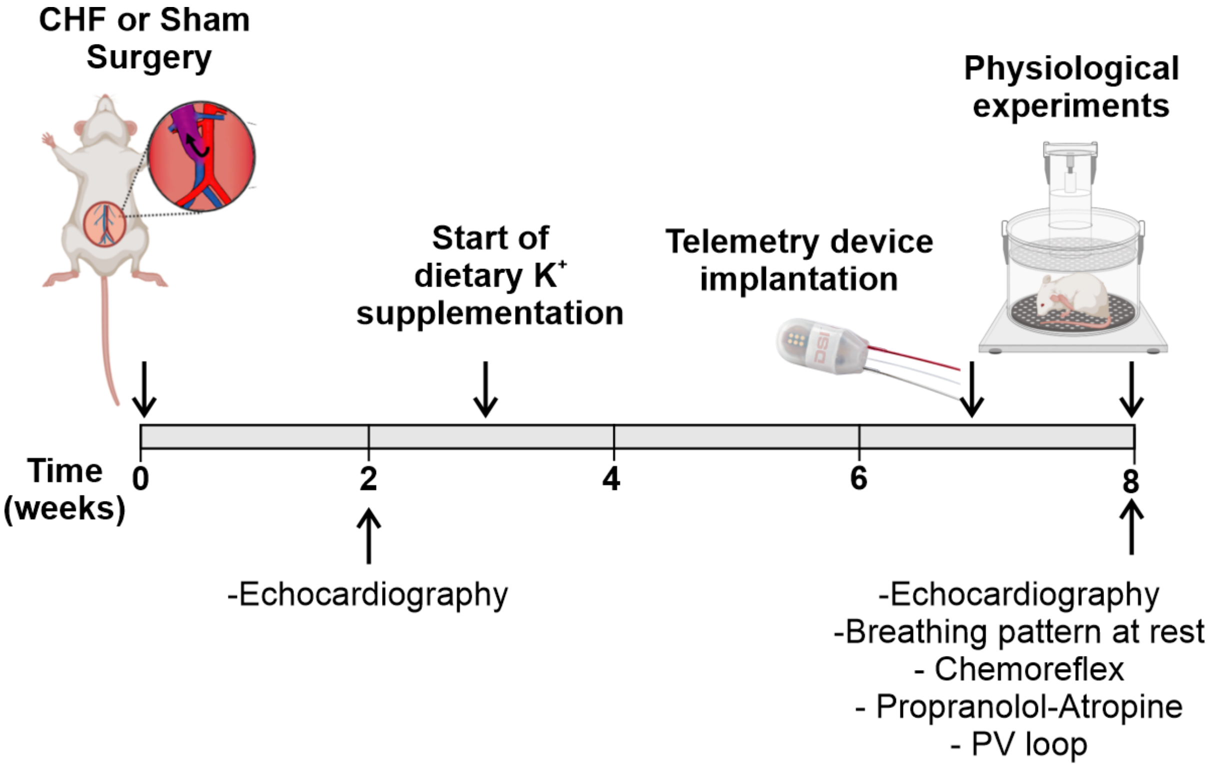

Figure S1. Experimental design. Adult male Sprague-Dawley rats underwent arteriovenous fistula to induce chronic heart failure (CHF) by volume overload. After 2 weeks, echocardiography was performed. Dietary K^+ supplementation started 3 weeks post-surgery and was maintained until the end of protocol. At week 7, a radiotelemetry device was implanted to measure blood pressure and heart rate in freely moving animals. At week 8 of the protocol, echocardiography, breathing, chemoreflex, autonomic and cardiac function were assessed.

Supplemental Tables

Table S1. Experimental diets composition**.**

	Control diet	Potassium-enriched diet
Nutrients		
**Protein, %**	22.5	22.5
Arginine, %	1.37	1.37
Cystine, %	0.30	0.30
Glycine, %	1.11	1.11
Histidine, %	0.53	0.53
Isoleucine, %	1.14	1.14
Leucine, %	1.68	1.68
Lysine, %	1.31	1.31
Methionine, %	0.49	0.49
Phenylalanine, %	1.00	1.00
Tyrosine, %	0.60	0.60
Threonine, %	0.83	0.83
Tryptophan, %	0.30	0.30
Valine, %	1.12	1.12
Serine, %	1.20	1.20
Aspartic Acid, %	2.35	2.35
Glutamic Acid, %	5.33	5.33
Alanine, %	1.17	1.17
Proline, %	1.73	1.73
Taurine, %	0.02	0.02

**Fat (ether extract), %**	5.4	5.4
**Fat (acid hydrolysis), %**	6.4	6.4
Cholesterol, ppm	195	195
Linoleic Acid, %	1.73	1.73
Linolenic Acid, %	0.16	0.16
Arachidonic Acid, %	0.00	0.00
Omega-3 Fatty Acids, %	0.34	0.34
Total Saturated Fatty Acids, %	1.75	1.75
Total Monounsaturated Fatty Acids, %	1.60	1.60
**Fiber (Crude), %**	4.0	4.0
Neutral Detergent Fiber, %	15.1	15.1
Acid Detergent Fiber, %	5.4	5.4
**Nitrogen-Free Extract, %**	52.0	52.0
Starch, %	30.4	30.4
Glucose, %	0.1	0.1
Fructose, %	0.2	0.2
Sucrose, %	1.0	1.0
Lactose, %	0.0	0.0
**Total Digestible Nutrients, %**	78.7	78.7
**Gross Energy, kcal/g**	4.10	4.10
**Physiological Fuel Value, kcal/g**	3.46	3.46
**Metabolizable Energy, kcal/g**	3.20	3.20
Minerals		
**Ash, %**	6.1	6.1
Calcium, %	1.00	1.00

Phosphorus, %	0.75	0.75
Phosphorus (non-phytate), %	0.44	0.44
Potassium, %	0.91	2.00
Magnesium, %	0.24	0.24
Sulfur, %	0.26	0.26
Sodium, %	0.26	0.26
Chlorine, %	0.44	0.44
Fluorine, ppm	16	16
Iron, ppm	380	380
Zinc, ppm	120	120
Manganese, ppm	96	96
Copper, ppm	12	12
Cobalt, ppm	0.27	0.27
Iodine, ppm	0.98	0.98
Chromium, ppm	1.4	1.4
Selenium, ppm	0.21	0.21
Vitamins		
Carotene, ppm	2.6	2.6
Vitamin K, ppm	1.9	1.9
Thiamin Hydrochloride, ppm	10	10
Riboflavin, ppm	14	14
Niacin, ppm	63	63
Pantothenic Acid, ppm	13	13
Choline Chloride, ppm	1600	1600
Folic Acid, ppm	1.2	1.2

Pyridoxine, ppm	7.6	7.6
Biotin, ppm	0.38	0.38
B_{12}, mcg/kg	75	75
Vitamin A, IU/g	29	29
Vitamin D_3, IU/g	2.4	2.4
Vitamin E, IU/kg	75	75

Table S2. Effect of K^+ supplementation on respiratory disorders incidence in CHF condition

	Sham (n=5)	CHF (n=5)	CHF+K^+ (n=5)
AHI (events/hr)	3.8 ± 0.7	8.4 ± 0.9 *	6.4 ± 0.5
Apnea (events/hr)	1.4 ± 0.4	4.8 ± 0.6 *	3.8 ± 0.4
Apnea Time (s)	2.3 ± 0.1	2.2 ± 0.2	2.6 ± 0.3
PSA (events/hr)	10.0 ± 2.0	10.8 ± 1.2	11.2 ± 0.9
PSA Time (s)	3.7 ± 0.2	3.9 ± 0.1 $^+$	2.9 ± 0.1
Hypopnea (events/hr)	2.4 ± 0.5	3.6 ± 0.5	3.0 ± 0.6
Hypopnea (s)	3.0 ± 0.1	2.6 ± 0.1	2.8 ± 0.3
Sigh (events/hr)	16.4 ± 1.2	17.2 ± 1.2 $^+$	13.4 ± 1.8

Values are expressed as mean ± S.E.M. AHI, apnea-hypoapnea index; PSA, post sigh apnea. *$p<0.05$ CHF *vs.* Sham, $^+p<0.05$ CHF *vs.* CHF+K^+

Table S3. Effects of K^+ supplementation in the diet on ventilatory parameters in CHF rats.

	Sham (n=5)	CHF (n=5)	CHF+K^+ (n=5)
**Normoxia**			
R_F (bpm)	77.2 ± 3.2	86.0 ± 4.3 *	80.0 ± 3.1
V_T (ml)	0.26 ± 0.02	0.27 ± 0.03	0.26 ± 0.04
V_E (ml/min)	20.4 ± 1.3	21.9 ± 1.4	19.2 ± 2.9
**Hypercapnia**			
R_F (bpm)	99.1 ± 2.8	139.0 ± 14.2 $^{*+}$	103 ± 1.4
V_T (ml)	0.4 ± 0.03	0.5 ± 0.04	0.3 ± 0.05
V_E (ml/min)	40.2 ± 4.7	60.8 ± 3.2 $^{*+}$	37.5 ± 6.2
**Hypoxia**			
R_F (bpm)	131.6 ± 5.6	139.8 ± 10.3	141.8 ± 6.2
V_T (ml)	0.3 ± 0.01	0.3 ± 0.03	0.3 ± 0.07
V_E (ml/min)	41.5 ± 2.3	44.7 ± 4.7	37.1 ± 10.3

Values are expressed as mean ± S.E.M. R_F, respiratory frequency; V_T, tidal volume; V_E, minute ventilation. *p<0.05 CHF *vs*. Sham, $^+$p<0.05 CHF *vs.* CHF+K^+.

Table S4. Arterial blood pressure and dietary K^+ supplementation in CHF

	Sham (n=5)	CHF (n=5)	CHF+K^+ (n=5)
MABP (mmHg)	103.1 ± 5.2	94.3 ± 3.5 *	85.6 ± 0.7
SBP (mmHg)	121.4 ± 7.1	110.0 ± 4.0	104.6 ± 0.9
DBP (mmHg)	89.8 ± 4.1	82.7 ± 3.5 *	72.0 ± 0.8
PP (mmHg)	31.5 ± 3.5	27.3 ± 2.8	32.6 ± 1.1
HR (bpm)	318.2 ± 8.1	271.3 ± 11.6 *+	331.9 ± 14.8

Values are expressed as mean ± S.E.M. MABP, mean arterial blood pressure; SBP systolic blood pressure; DBP, diastolic blood pressure; PP, pulse pressure; HR, heart rate. *$p<0.05$ CHF *vs.* Sham, $^+p<0.05$ CHF *vs.* CHF+K^+.

Table S5. Echocardiography parameters

	Sham (n=5)		CHF (n=5)		$CHF+K^+$ (n=5)	
	preDiet	postDiet	preDiet	postDiet	preDiet	postDiet
LVEDV (µl)	218.0 ± 20.6	201.3 ± 14.9	290.1 ± 13.2 $^+$	369.7 ± 24.7 *‡	311.0 ± 19.5	402.0 ± 23.8 *
LVESV (µl)	47.4 ± 6.1	37.2 ± 6.8	94.6 ± 12.0 $^+$	124.7 ± 14.3 *‡	91.1 ± 14.0	140.3 ± 22.3 *
SV (µl)	170.6 ± 24.0	164.1 ± 11.3	195.4 ± 9.5 $^+$	245.0 ± 12.9 *‡	219.9 ± 12.3	261.7 ± 14.6 *
EF (%)	76.5 ± 5.1	81.9 ± 2.5 *	67.6 ± 3.1 $^+$	66.6 ± 2.0 ‡	71.1 ± 3.1	65.6 ± 4.0
FS (%)	50.2 ± 3.1	52.7 ± 2.7	39.1 ± 2.5 $^+$	35.2 ± 1.7 ‡	42.1 ± 2.7	40.9 ± 3.7
BW (g)	372.2 ± 8.2	437.0 ± 5.4	373.0 ± 10.6	425.8 ± 16.6	386 ± 17.9	436.3 ± 13.4
HW (g)	-	1.3 ± 0.0	-	1.8 ± 0.1 ‡	-	1.6 ± 0.0
HW/BW (mg/g)	-	2.8 ± 0.2	-	3.7 ± 0.2 ‡	-	3.8 ± 0.2

Values are expressed as mean ± S.E.M. LVEDV, left ventricle end diastolic volume; LVESV, left ventricle end systolic volume; SV, stroke volume; EF, ejection fraction; FS, fractional shortening; HW/BW, heart weight/body weight ratio. *$p<0.05$ preDiet *vs.* postDiet, $^+p<0.05$ CHF preDiet *vs.* Sham preDiet, $^‡p<0.05$ CHF postDiet *vs.* Sham postDiet.

Table S6. Effect of K^+ supplemented diet on intraventricular cardiac parameters.

	Sham (n = 5)	CHF (n = 5)	CHF+K^+ (n = 5)
**Global Cardiac Function**			
HR, beats/min	373.0 ± 23.41	335.40 ± 32.81	338.81 ± 30.70
CO, ml/min	63.39 ± 10.55	94.61 ± 8.60*	87.12 ± 15.60
LVEDP, mmHg	2.71 ± 0.22	5.40 ± 0.54*	4.31 ± 0.20*
**Preload and Afterload**			
LVEDV, µl	251.61 ± 28.50	383.10 ± 19.51*	378.20 ± 42.01*
Ea, mmHg/µl	0.56 ± 0.06	0.31 ± 0.02*	0.46 ± 0.15
**Systolic left ventricular function**			
LVEF, %	75.57 ± 5.34	81.66 ± 4.63	71.43 ± 8.46
LVESV, µl	82.51 ± 8.57	100.41 ± 17.24	108.98 ± 9.95
dp/dt_{max}, mmHg/s	15,077.20 ± 4,810.53	8,685.40 ± 781.57	8,977.50 ± 1,561.98
**Diastolic left ventricular function**			
Active relaxation			
Tau, ms	6.97 ± 0.75	10.15 ± 1.12	9.87 ± 1.63
dp/dt_{min}, mmHg/s	-6,609.40 ± 824.71	-5,171.40 ± 414.99	-4,543.75 ± 558.18

Values are expressed as mean ± S.E.M. HR: heart rate; CO, cardiac output; LV, left ventricular; EDP, end diastolic pressure; EDV, end diastolic volume; Ea, arterial elastance; EF, ejection fraction; ESV, end systolic volume; Tau, time constant of relaxation. One way ANOVA, follow Sidak *post hoc* analysis. *$p<0.05$ CHF *vs*. Sham.
